# Gemcitabine-induced β4 integrin drives cancer progression and gemcitabine resistance in pancreatic cancer

**DOI:** 10.1186/s13046-026-03718-2

**Published:** 2026-04-24

**Authors:** Yoshinobu Kariya, Haruto Suzuki, Yukiko Kariya, Michiru Nishita

**Affiliations:** Department of Biochemistry, Fukushima Medical University School of Medicine, 1 Hikarigaoka, Fukushima City, Fukushima 960-1295 Japan

**Keywords:** PDAC, β4 integrin, Gemcitabine, Drug resistance, Cancer progression, Epigenetic, β-catenin, Laminin-332, Src, Histone acetylation

## Abstract

**Background:**

Pancreatic ductal adenocarcinoma (PDAC) is characterized by high mortality and the frequent emergence of chemoresistance, particularly to gemcitabine (GEM). Recent studies have indicated that tumors exhibit increased malignancy following the acquisition of chemoresistance. However, the underlying molecular mechanisms remain poorly understood. This study aimed to elucidate the mechanisms by which PDAC cells acquire GEM resistance and how this resistance drives cancer progression, focusing on identifying the key molecular drivers involved in these processes.

**Methods:**

We established GEM-resistant PDAC cell lines (GEM-Panc-1 and GEM-MIA PaCa-2) and characterized their malignant phenotypes using migration, invasion, proliferation, and sphere-formation assays, as well as *in vivo* models for tumor growth, tumor initiation, and metastasis. The functional role of β4 integrin (encoded by *ITGB4*) was evaluated using stable knockdown and overexpression systems. To investigate the regulatory mechanisms, we employed RNA sequencing, chromatin immunoprecipitation (ChIP), and promoter reporter assays. Clinical relevance was assessed using TCGA datasets and Kaplan‒Meier analysis.

**Results:**

GEM-resistant PDAC cells exhibited enhanced invasive potential, tumorigenicity, and stemness, which coincided with the upregulation of β4 integrin. Knockdown of β4 integrin in GEM-resistant cells attenuated these malignant properties and partially restored GEM sensitivity, whereas its ectopic expression in parental cells conferred aggressive phenotypes. Clinically, high expression of *ITGB4* and its ligand, the laminin-332 subunit genes (*LAMA3*, *LAMB3*, and *LAMC2*), correlated with poor overall survival in PDAC patients. Mechanistically, the β4 integrin/laminin-332 axis promoted cell motility and GEM resistance via Src activation. Furthermore, acquired GEM resistance epigenetically supports the activation of *ITGB4* transcription, which is associated with β-catenin nuclear translocation and p300-mediated histone H3 acetylation (H3K27ac) at the *ITGB4* promoter.

**Conclusions:**

Our findings suggest that GEM-induced β4 integrin expression, potentially supported by β-catenin/p300-mediated epigenetic remodeling, is a critical determinant of GEM resistance and PDAC progression. Targeting the β4 integrin/laminin-332/Src signaling axis may provide a promising therapeutic strategy to overcome chemoresistance and improve clinical outcomes in patients with advanced PDAC.

**Supplementary Information:**

The online version contains supplementary material available at 10.1186/s13046-026-03718-2.

## Background

Pancreatic ductal adenocarcinoma (PDAC) accounts for approximately 90% of all pancreatic malignancies and is the leading cause of cancer-related mortality worldwide, with a 5-year overall survival rate of less than 10% [1, 2]. The incidence of PDAC is increasing, and it is projected to become the second leading cause of cancer-related mortality by 2030 [2]. Nearly all PDAC patients receive chemotherapy with anticancer agents, including gemcitabine (GEM), regardless of surgical eligibility [3, 4]. The majority of PDAC patients who undergo chemotherapy develop resistance to therapy. Additionally, more than 80% of all patients experience recurrence within 2 years, and the prognosis for patients with recurrent PDAC is poor, making subsequent treatment challenging [4–6]. Therefore, a deeper understanding of the molecular mechanisms underlying the development of chemoresistance and recurrence in patients with PDAC is crucial for improving the survival rate of patients with PDAC.

A recent clinical study revealed that the development of chemoresistance worsens the prognosis of patients with PDAC receiving adjuvant GEM therapy after surgery [7]. Furthermore, chemotherapy promotes metastasis by increasing the number of circulating tumor cells [8]. These findings contradict the conventional concept that distant metastases occurring after successful treatment originate from micrometastases that exist prior to treatment [4, 8]. Chemotherapy also promotes the selection or generation of resistant and metastatic cancer stem cells (CSCs) at the primary site [9] and alters the tumor microenvironment and energy metabolism [8, 10, 11]. Thus, these studies suggest that chemotherapy itself may induce cancer progression, but the molecular mechanisms involved remain poorly understood.

In this study, we aimed to understand how chemotherapy induces cancer progression in PDAC cells. We found that GEM upregulated β4 integrin (encoded by *ITGB4*) expression, thereby conferring GEM resistance and malignant properties to PDAC cells. Mechanistically, GEM upregulated β-catenin expression and nuclear translocation, which was accompanied by histone H3 acetylation at the *ITGB4* promoter site, potentially contributing to the activation of *ITGB4* transcription.

## Methods

### Reagents

Gemcitabine hydrochloride (#073–06631), doxorubicin hydrochloride (#040–21521), and paclitaxel (#167–28161) were obtained from WAKO. Dasatinib and saracatinib, both Src inhibitors, were purchased from Santa Cruz (#sc-358114) and Focus Biomolecules (#10-2901), respectively. The p300 histone acetyltransferase inhibitor C646 was obtained from AdipoGen (#AG-CR1-3508-M001). IQ1, a specific inhibitor of the p300/β-catenin interaction, was obtained from MedChemExpress (#HY-10593). The β-catenin/CBP interaction inhibitor PRI-724 was purchased from Abcam (#ab229168). Dimethyl sulfoxide (DMSO) was obtained from Nacalai Tesque (#13407-45). Human purified plasma fibronectin (#FC010) and mouse laminin-111 (#L2020) were purchased from Millipore. Human laminin-511/521 was obtained from Gibco Life Technologies (#12163-010).

### Cell culture

The human PDAC cell lines Panc-1 and MIA PaCa-2, the human gastric cancer cell line MKN45, and the human embryonic kidney cell line 293T were obtained from the RIKEN BRC through the National Bio-Resource Project of the MEXT, Japan. Modified human 293 phoenix cells were a gift from Dr. M. Peter Marinkovich (Stanford University, Stanford, CA). The cells were expanded within 3 passages after purchase, and multiple lots were stored at − 150°C. GEM-Panc-1 and GEM-MIA PaCa-2 cells were generated via stepwise exposure to increasing concentrations of GEM (from 10 nM to 1200 nM for Panc-1 cells; from 5 nM to 50 nM for MIA PaCa-2 cells). GEM-MIA PaCa-2 cells were also generated via exposure to 20 nM GEM. Doxorubicin- and paclitaxel-resistant Panc-1 cells were generated via stepwise exposure to increasing concentrations of doxorubicin (from 5 nM to 1100 nM) and paclitaxel (from 5 nM to 800 nM), respectively. 293T, 293 phoenix, and MIA PaCa-2 cells were cultured in DMEM supplemented with 10% FBS and penicillin‒streptomycin sulfate. Panc-1 cells were cultured in RPMI 1640 supplemented with 1 mM sodium pyruvate, 2.5 g/L D (+)-glucose, 10 mM HEPES, 10% FBS, and penicillin‒streptomycin sulfate. MKN45 cells were maintained in RPMI 1640 supplemented with 10% FBS and penicillin‒streptomycin sulfate. All cells were maintained in low-passage culture after purchase or receiving them, with the exception of drug-resistant cells, and were tested for mycoplasma contamination. The cell morphology was observed under an IX71 phase contrast microscope (Olympus), and photographs were taken.

### Genetic knockdown and ectopic expression

To achieve stable knockdown of β4 integrin, GEM-Panc-1 cells were transduced with lentiviral vectors encoding shRNAs targeting human β4 integrin (#TRCN0000057768 and #TRCN0000057771, Dharmacon) and control shRNA (#RHS4459, Dharmacon) via the Trans-Lentiviral shRNA Packaging Kit (#TLP5912, GE Healthcare) according to the manufacturer’s instructions. To establish stable gene-overexpressing cell lines, cells were transduced with LZRS-blast retroviral vectors encoding wild-type (WT), adhesion-deficient (AD), or cytoplasmic domain-deleted (Δcyto) β4 integrin [12], as well as β-catenin. To prepare control cells, the cells were transduced with LZRS-blast retroviral vectors encoding lacZ. Retroviral vectors were transfected into 293 phoenix cells via FuGene6 reagent (#11814443001, Roche). The cells were selected with 5 µg/mL puromycin (#P8833, Sigma‒Aldrich) for 2 weeks, after which the retrovirus was produced in 293 phoenix cells. One day before infection, 5 × 10^4^ cells were plated in a 6-well plate (#353046, BD Falcon). After incubation with 5 µg/mL polybrene (#10768-9, Sigma‒Aldrich) for 15 min, the media were exchanged with 3 mL of retroviral supernatant containing 5 µg/mL polybrene. The plates were centrifuged at 200 × g for 1 h at 32°C via a Hitachi CR22N centrifuge machine with an R5S4 rotor, and the viral supernatant was replaced with fresh growth media. To establish a stable cell line, cells were selected with 10 µg/mL blasticidin S (#203350, Calbiochem) for retroviral infection and 5 µg/mL puromycin for lentiviral infection. Dicer-substrate small interfering RNAs (DsiRNAs) for targeted genes (*CTNNB1* and *EP300*) were purchased from IDT. The DsiRNA sequences used for targeting are as follows:

CTNNB1 si#1 (hs.Ri.CTNNB1.13.1): 5’- CACAACCUUUUAUUACAUCAAGAAG-3’,

5’ -CUUCUUGAUGUAAUAAAAGGUUGUGGA-3’;

CTNNB1 si#2 (hs.Ri.CTNNB1.13.2): 5’-UGAAUACUGCUACAGCAAUUUCUAA-3’,

5’ -UUAGAAAUUGCUGUAGCAGUAUUCACU-3’;

EP300 si#1 (hs.Ri.EP300.13.2): 5’-GGUUUAAAGCAAACAUGCAAGAUGA-3’,

5’-UCAUCUUGCAUGUUUGCUUUAAACCAC − 3’;

EP300 si#2 (hs.Ri.EP300.13.8): 5’- GAGGGAUGAUAGAAUACAAAGAATA-3’,

5’ -UAUUCUUUGUAUUCUAUCAUCCCUCAG-3’.

One day before transfection, 1 × 10^6^ cells were seeded in 2.5 mL of growth medium per well in a 6-well plate (#353046, BD Falcon). The cells were transfected with DsiRNA complexes targeting *CTNNB1* (using *Trans*IT-siQUEST; #MIR 2114, Mirus Bio) or *EP300* (using Lipofectamine RNAiMAX Transfection Reagent; #13778-100, Thermo Fisher Scientific). Cells were then incubated for 48 h and 72 h, respectively, prior to analysis. A negative control DsiRNA (#51-01-14-03, IDT) was used as a reference.

### Animal studies

All the animal studies were performed in accordance with protocols approved by the Fukushima Medical University Animal Care and Use Committee. During the experiment, the mice were examined for clinical symptoms and evidence of toxicity. This included observing their food and water intake, walking ability, weight loss, and presence of ulceration or necrosis in their tumors. For the tumorigenicity assay, 1 × 10^6^ cells in 100 µL PBS were subcutaneously injected along with 100 µL Matrigel (#354234, BD Biosciences) into 6-week-old female nude mice (BALB/c nu/nu, SLC). Primary tumor growth was measured once a week with a caliper, and the volume was calculated using the formula D (long diameter) × d (short diameter)^2^ × 1/2. At the end of each experiment, all the nude mice were euthanized. The tumors were carefully dissected, weighed, and processed for immunohistochemical analyses. For the metastasis assay, 1 × 10^6^ cells in 100 µL PBS were injected into the tail vein of 6-week-old female nude mice. The mice were euthanized at 6 weeks after the injection, and their lungs were removed and fixed in 4% paraformaldehyde/PBS. The fixed tissues were embedded in paraffin, sectioned, and stained with Mayer’s hematoxylin and eosin (H&E). Images were captured using a Nanozoomer-SQ (#C13140–L04, Hamamatsu) and analyzed using NDPview2 software. For the *in vivo* tumor-initiating assay, Panc-1 and GEM-Panc-1 cells were suspended in 100 µL PBS and subcutaneously injected with 100 µL Matrigel into 6-week-old female nude mice in a limiting dilution series of 1 × 10^6^, 1 × 10^4^, and 1 × 10^3^ cells per injection (*n* = 5 for each dose). The presence or absence of tumors was monitored for 6 weeks after injection. The frequency of tumor-initiating cells and the statistical significance between groups were calculated using the Extreme Limiting Dilution Analysis (ELDA) online tool (http://bioinf.wehi.edu.au/software/elda/) [13]. The single-hit model was validated by the likelihood ratio test (*P* = 0.298).

### Immunohistochemistry

The tissue Sections (2.5 μm) were deparaffinized with xylene and rehydrated in decreasing concentrations of ethanol. For Ki-67 staining, antigen retrieval was performed via microwave treatment in Tris-EDTA buffer (10 mM Tris-base, 1 mM EDTA, 0.05% Tween 20, pH 9.0) for 15 min. The tissue slides were allowed to cool for 20 min in Tris-EDTA buffer and then immersed in 0.3% H_2_O_2_ in methanol (v/v) for 20 min at room temperature to inactivate endogenous peroxidase. After being rinsed three times in PBS for 5 min, the samples were incubated with anti-Ki-67 (SP6, #RMAB004, Diagnostic BioSystems, 1:200) in 2% BSA/PBS overnight at 4°C. The sections were incubated with peroxidase-labeled secondary antibodies [Histofine simple stain MAX‒PO (MULTI) Kit, #424152, Nichirei Corp] for 30 min at room temperature. After the samples were washed three times with PBS for 5 min, peroxidase activity was visualized using a Histofine DAB substrate Kit (#425011, Nichirei Corp.). The slides were then counterstained with Meyer’s hematoxylin solution (#131–09665, WAKO), dehydrated and mounted with mounting media (Mount-Quick, #DM-01, Daido Sangyo). Images were captured using a Nanozoomer-SQ and analyzed using a NDPview2 software. Five randomly chosen fields were selected per sample, and the Ki-67-positive and negative cells were counted. At least 140 cells were counted per field.

### Preparation of deposited extracellular matrix (ECM) and conditioned medium, and purification of laminin-332

To prepare the deposited ECM, cells were cultured in growth medium for five days, with the medium changed daily. The cells were then washed twice with PBS and detached from the plates by incubation with 10 mM EDTA. After two additional PBS washes, the plates were treated twice with 20 mM NH_4_OH for 5 min at room temperature to ensure complete decellularization. After five PBS washes, the plates were examined under a microscope to confirm the absence of cellular debris. Finally, the deposited ECM was recovered from the plates using a cell scraper in SDS sample buffer [62.5 mM Tris-HCl (pH6.8), 2% (w/v) SDS, 10% (w/v) glycerol. 0.005% (w/v) bromophenol blue]. For the preparation of conditioned medium, the cells were grown to confluence in growth medium, washed twice with PBS, and then incubated in serum-free medium. The serum-free medium was collected every 2 or 3 days and centrifuged at 190 × g for 10 min at 4°C to remove debris, and the resulting supernatant was used as the conditioned medium. For Western blot analysis of secreted and deposited proteins, equal volumes of conditioned medium or ECM samples harvested from 90 mm dishes were loaded into each lane. Cell numbers were determined at the time of harvest to evaluate the production of laminin-332 relative to cell density. For the purification of laminin-332, the protein in the conditioned medium from MKN45 cells was precipitated with 80% saturated ammonium sulfate, and then, the precipitate was dissolved and dialyzed against gelatin column buffer [20 mM Tris-HCl (pH 7.5), 0.1 M NaCl, 0.005% Brij 35, 0.1% CHAPS] overnight at 4°C. The samples were centrifuged at 39,900 × g for 30 min at 4 °C to remove the undissolved protein, the precleared solution was applied to a gelatin column, and the flow-through was applied to an anti-laminin γ2 antibody (clone D4B5, #MAB19562, Millipore) column. After washing with the antibody column buffer [20 mM Tris-HCl (pH 7.5), 0.5 M NaCl, 0.005% Brij 35, 0.1% CHAPS], followed by Milli-Q water, the binding laminin-332 proteins were eluted with 0.05% trifluoroacetic acid. The eluted solution was immediately neutralized with Tris-HCl (pH 8.0), and Brij35 and CHAPS were added to final concentrations of 0.005% and 0.1%, respectively.

### Cell migration and invasion assays

Cell migration and invasion assays were performed using 24-well chemotaxis chambers (Cell culture companion plates #353504 and 8.0-µm inserts #353097, BD Transduction Laboratories). For the substrate-mediated migration assay, chamber inserts were coated on the bottom side with 300 µL of the indicated purified substrate at 4°C for 16 h and then blocked with PBS containing 1.2% BSA at 37°C for 1 h. For the invasion assay, chamber inserts were coated on the upper side with 100 µL of 1.6 mg/mL Matrigel (#354234, BD Transduction Laboratories). A total of 1 × 10^5^ cells (migration assay: 200 µL of serum-free medium; invasion assay: 200 µL of medium containing 0.5% FBS) were seeded into the chamber inserts and 750 µL of serum-containing medium (noncoated assays: 10% FBS; substrate-coated assays: 1% FBS) was added to the lower chamber. For the inhibition assay, the cells were incubated with dasatinib (200 nM) or saracatinib (1 µM) for 20 min at room temperature before plating. After 4 h (for the migration assay of Panc-1 cells) or 22 h (for the migration assay of MIA PaCa-2 cells and the invasion assay of Panc-1 and MIA PaCa-2 cells), the cells were fixed with 2.5% glutaraldehyde for 10 min and stained with 0.5% crystal violet in 20% methanol for 20 min. The cells and the Matrigel on the upper side of the membrane were removed with cotton swabs. Three randomly chosen fields on the lower side of the membrane were photographed using an IX71 phase contrast microscope (Olympus), and the migrated cells were counted.

### Cell proliferation assay

The cells were seeded into 35 mm dishes in 2 mL of growth medium and incubated at 37°C in a 5% CO_2_ incubator. After being washed twice with PBS, the grown cells were harvested with 0.25% (w/v) trypsin/1 mM EDTA 4Na (#201–16945, WAKO), and the cell numbers were counted using a hemocytometer.

### Cell viability assay

The cells were plated at a density of 5 × 10^3^ cells per well in 96-well microplate (#353072, BD FALCON) with 100 µL of growth medium and incubated at 37°C in a 5% CO_2_ incubator. After incubation for 24 h, the growth medium was replaced with growth medium with or without drugs, and the cells were further incubated at 37°C in the presence of 5% CO_2_. After incubation for 72 h, the medium was replaced with 100 µL of growth medium with 10 µL of Cell Counting Kit-8 solution (#343–07623, DOJINDO). Each well of the plate was then further incubated at 37°C in a 5% CO_2_ incubator for 1.5 h for MIA PaCa-2 cells and 2.5 h for Panc-1 cells. The color intensity was subsequently measured at 450 nm using a microplate reader (model 680, Bio-Rad). The relative survival rate in the presence of the indicated drug was then normalized to the control set to 100%. The *IC*_50_ values were calculated using GraphPad Prism Version 6.

### Sphere assay

For the *in vitro* sphere assay, cells were plated at a density of 1 × 10^4^ cells per well in a 96-well ultra-low attachment microplate (#3474, Corning) with 100 µL of serum-free medium containing B-27 supplement (#17504, Invitrogen, 1:50), 20 ng/mL human epidermal growth factor (#E9644, Sigma), 20 ng/mL human basic fibroblast growth factor (#064–04541, WAKO), and penicillin/streptomycin sulfate. Colonies with a diameter greater than 100 μm for Panc-1 cells and 200 μm for MIA PaCa-2 cells were counted after incubation for 4 days at 37°C in a 5% CO_2_ incubator.

### FACS

To analyze the cell surface expression of integrins, the cells were detached from a dish with 0.25% (w/v) trypsin/1 mM EDTA 4Na. After trypsinization was quenched with medium containing 10% FBS, the cells were washed twice with PBS containing 1 mM EDTA (PBS/EDTA) and then suspended in PBS/EDTA. The cells were then incubated with each integrin-specific monoclonal antibody on ice for 30 min. After being washed once with PBS/EDTA, the cells were incubated with an Alexa Fluor 488-conjugated goat secondary anti-mouse IgG antibody (#A11029, Invitrogen, 1:500) or an Alexa Fluor 546-conjugated goat secondary anti-rat IgG antibody (#A11081, Invitrogen, 1:500). After incubation on ice for 15 min, the cells were washed three times with PBS/EDTA and then analyzed via flow cytometry using a FACSCalibur and a CellQuest software (BD Biosciences). The primary antibodies used for flow cytometry were as follows: α3 integrin (clone P1B5, #sc-13545, Santa Cruz Biotechnology, 1:100), α5 integrin (clone P1D6, #921704, BioLegend, 1:100), α6 integrin (clone GoH3, #sc-19622, Santa Cruz Biotechnology, 1:100), β1 integrin (clone P5D2, #sc-13590, Santa Cruz Biotechnology, 1:100), β4 integrin (clone 58XB4, #327802, BioLegend, 1:100), and αvβ3 integrin (clone 23C6, #sc-7312, Santa Cruz Biotechnology, 1:100).

### Western blot

To prepare cell lysates, the cells were washed twice with ice-cold PBS and then lysed with lysis buffer [1% NP40, 25 mM Tris-HCl (pH 7.5), 150 mM NaCl, 5 mM EDTA, 1% sodium deoxycholate, 0.1% SDS, protease inhibitor cocktail (#25955-24, Nacalai Tesque, 1:100), and phosphatase inhibitor cocktail (#07575-51, Nacalai Tesque, 1:100)]. After incubation for 20 min on ice, the cell lysates were clarified by centrifugation at 20,400 × g for 20 min at 4°C, and the resulting supernatant was used as a cell lysate sample. The protein concentration of each cell lysate sample was determined using a Protein Assay Kit (#29449-44, Nacalai Tesque). For Western blot, proteins were separated on SDS‒PAGE gels under reducing conditions and then transferred to nitrocellulose membranes (#037-25653 and #033-22453, WAKO). The membranes were blocked with 5% skim milk in TBS with 0.1% (v/v) Tween 20 (TBST) for 1 h at room temperature, washed three times with TBST for 5 min, and probed with primary antibodies for 1 h at room temperature or overnight at 4°C. Primary antibodies against the following proteins were used: β4 integrin (clone H101, #sc-9090, Santa Cruz Biotechnology, 1:1,000), α-tubulin (clone DM 1A, #T9026, SIGMA, 1:10,000), phospho-Src (Tyr416) (#2101, Cell Signaling Technology, 1:1,000), phospho-Src (Tyr416) (D49G4, #6943, Cell Signaling Technology, 1:1,000), phospho-Src (Tyr530) (#2105, Cell Signaling Technology, 1:1,000), c-Src (clone SRC2, #sc-18, Santa Cruz Biotechnology, 1:1,000), E-cadherin (clone 36/E-Cadherin, #610182, BD Transduction Laboratories, 1:1,000), N-cadherin (clone 32/N-Cadherin, #610920, BD Transduction Laboratories, 1:1,000), vimentin (clone V9, #sc-6260, Santa Cruz Biotechnology, 1:1,000), ZEB1 (clone D80D3, #3396, Cell Signaling Technology, 1:1,000), cytokeratin 18 (clone RGE53, #MAB3234, Millipore, 1:1,000), β-catenin (clone 14/Beta-Catenin, #610154, BD Transduction Laboratories, 1:1,000), p300 (clone D2×6N, #54062, Cell Signaling Technology, 1:1,000), lamin B1 (#CAF548Hu01, Cloud-Clone Corp, 1:5,000), CD133 (#66666-1-Ig, proteintech, 1:2,000), CD44 (clone IM7, #103001, BioLegend, 1:1,000), laminin-β3 (clone 17/Kalinin B1, #610423, BD Transduction Laboratories, 1:5,000), Acetyl-Histone H3 (Lys27) (clone D5E4, #8173, Cell Signaling Technology, 1:1,000), and Histone H3 (clone 1B1-B2, #819411, BioLegend, 1:10,000). The membranes were washed three times with TBST for 5 min each and incubated with the following horseradish peroxidase-conjugated secondary antibodies for 1 h at room temperature: anti-mouse IgG (#7076, Cell Signaling Technology, 1:5,000), anti-rabbit IgG (#W401B, Promega, 1:5,000 or #7074, Cell Signaling Technology, 1:5,000), or anti-rat IgG (#62-9520, Thermo Fisher Scientific, 1:1,000) antibodies. After washing three times with TBST for 5 min, signals were detected using a Trident femto-ECL reagent (#GTX14698, GeneTex) and visualized using Imager and Image Saver software (#AE-9300H-CP, ATTO). Densitometry was performed using Image J.JS software (https://ij.imjoy.io).

### RT‒qPCR

Total RNA was isolated using the NucleoSpin RNA Plus Kit (#740984.50, Macherey-Nagel). Five hundred µg of total RNA was used for cDNA synthesis using the PrimeScript RT Master Mix Kit (RR036A, Takara). Quantitative PCR (qPCR) was performed on a StepOne PCR machine (Thermo Fisher Scientific) using PrimeTime Gene Expression Master Mix (#1055770, IDT) and primers from the PrimeTime Mini qPCR Assay system (IDT) according to the manufacturer’s instructions. The data were collected and analyzed using StepOne software. All the mRNA quantification data were normalized to those of HPRT1. The sequences of primers used were as follows: β4 integrin forward, 5’- CTGCTACTGCTGCTATGCT-3’; β4 integrin reverse, 5’- CAGCATGTAGTGGTCTTCCTT-3’; HPRT1 forward, 5’- GCGATGTCAATAGGACTCCAG-3’; and HPRT1 reverse, 5’- TTGTTGTAGGATATGCCCTTGA-3’.

### Chromatin immunoprecipitation (ChIP)-qPCR

For the ChIP assay, 1 × 10^7^ cells were incubated with 37% formaldehyde (#252549, Sigma‒Aldrich) diluted to a final concentration of 1% in a rotator for 5 min at room temperature, and a 1/5 volume of 1.25 M glycine was added to quench the formaldehyde. The samples were further incubated in a rotator for 5 min at room temperature and then centrifuged at 190 × g for 5 min at 4°C, after which the media was removed. The cell pellet was washed twice with cold PBS, resuspended in 500 µL of NP40 lysis buffer [0.5% NP40, 10 mM Tris-HCl (pH 7.5), 10 mM NaCl, and protease inhibitor cocktail (1:100)], and centrifuged at 7,300 × g for 2 min at 4°C. After removal of the supernatant, the cell pellet was resuspended in 500 µL of NP40 lysis buffer, incubated on ice for 5 min, and then suspended in 100 µL of SDS lysis buffer [50 mM Tris-HCl (pH 8.0), 10 mM EDTA (pH 8.0), 1% SDS, and protease inhibitor cocktail (1:100)]. After incubation on ice for 10 min, the sample was mixed with 400 µL of 1% Triton X-100 lysis buffer [1% Triton X-100, 15 mM Tris-HCl (pH 8.0), 1 mM EDTA (pH 8.0), 150 mM NaCl, and protease inhibitor cocktail (1:100)] and then sonicated at 40% amplitude (10 sec on, 50 sec off, for a total of 5 min) and 50% amplitude (10 sec on, 50 sec off, for a total of 10 min) using a Vibra-Cell VC 130 PB ultrasonic processor (Sonics & Materials). The sample was centrifuged at 20,400 × g for 5 min at 4°C, and the supernatant was added to 600 µL of 1% Triton-X100 lysis buffer. For input, 100 µL of the sample containing 0.5% SDS and 0.25 µg/µL RNase A (#30100-31, Nacalai Tesque) was incubated at 65°C. After incubation for 6 h, proteinase K (#10012, Cell Signaling Technology) was added to the sample to a final concentration of 0.56 µg/µL, and the lysates were further incubated at 56°C overnight. DNA was purified using a QIAquick PCR Purification Kit (#28104, QIAGEN). ChIP was performed using a rabbit monoclonal anti-Acetyl-Histone H3 (Lys27) antibody (clone D5E4, #8173, Cell Signaling Technology, 1:100) or a normal rabbit IgG (#CR1, Sino Biological) as a negative control and Dynabeads M-280 sheep anti-rabbit IgG (#11203D, Thermo Fisher Scientific) overnight at 4°C. Immunoprecipitated beads were washed with low-salt buffer [1% Triton X-100, 20 mM Tris-HCl (pH 8.0), 2 mM EDTA (pH 8.0), 150 mM NaCl, 0.1% SDS], followed by high-salt buffer [1% Triton X-100, 20 mM Tris-HCl (pH 8.0), 2 mM EDTA (pH 8.0), 500 mM NaCl, 0.1% SDS], LiCl buffer [1% NP40, 10 mM Tris-HCl (pH 8.0), 1 mM EDTA (pH 8.0), 25 mM LiCl, 1% sodium deoxycholate], and TE buffer [10 mM Tris-HCl (pH 8.0), 1 mM EDTA (pH 8.0)]. The beads were then incubated at 65°C in salting method buffer [20 mM Tris-HCl (pH 8.0), 10 mM EDTA (pH 8.0), 400 mM NaCl, 0.5% SDS] containing 240 ng/µL RNase A. After incubation for 6 h, proteinase K was added to the sample to a final concentration of 570 ng/µL, and the beads were further incubated at 56°C overnight. DNA was purified using a QIAquick PCR Purification Kit. The recovered DNA was quantified by qPCR using THUNDERBIRD Next SYBR qPCR Mix (#QPX-201, TOYOBO) with specific primers according to the manufacturer’s instructions. The sequences of primers used for the *ITGB4* promoter are hITGB4 ChIP Fwd, 5’- CATGGTGCTAGGTGCTAGAGAGTAGCTG − 3’ and hITGB4 ChIP Rev, 5’- AGTTTATCCTTCTTGTCCTTGAAGACGTTG − 3’ [14]; those for the *LAMC2* promoter are LAMC2 promoter F, 5’-CTCCGGGGAATCTCGCACA-3’; and those for the LAMC2 promoter R, 5’- CACAAACCGGGCTGGAAAATC-3’ [15]. All the qPCRs were performed in three independent experiments conducted in triplicate, and the results were calculated as a percentage of the input DNA using the following formula: Input percentage = 2 ^[(CT value of input−log2 (dilution factor)) − (CT value of IP)]^ ×100. The dilution factor = initial volume of sample lysate used in the IP pathway/initial volume of sample lysate used in the input pathway.

### Nuclear and cytoplasmic fractionation

The preparation of nuclear and cytoplasmic extracts was performed using a LysoPure Nuclear and Cytoplasmic Extraction Kit (#295-73901, Wako) according to the manufacturer’s protocol.

### Dual-luciferase reporter assay

The *ITGB4* promoter region was amplified by PCR using KOD -Plus- (#KOD-201, TOYOBO) with the following primers: ITGB4-reporter assay template-F: 5’-CTGAGCTCGCTAGCCGAAGCCCCCTATGAGCCAAC-3’; and ITGB4-reporter assay template-R: 5’-CAGTACCGGATTGCCAGGGGAAGGGGAGACAAAAGC-3’. The amplified PCR fragment was cloned into the *Xho* I-*Hind* III site of pGL4.27 [*luc2P*/minP/Hygro] firefly luciferase reporter vector (#E845A, Promega) using NEBuilder HiFi DNA Assembly Master Mix (#E2621S, NEB). The resultant plasmid contained no minimal promoter (minP). Subsequently, the plasmid was digested with *Nhe* I and self-ligated, and this construct was designated as *ITGB4* promoter (Chr 17: 75721260–75721690). As a negative control, a pGL4.27 vector lacking the minP (designated as pGL4.27 ΔminP) was prepared by inverse PCR using KOD -Plus- and the following primers: minP del-F for pGL4.27: 5’-TCGGCGGCCAAGCTTGGCAATCCGGTACTGTTGG-3’; and minP del-R for pGL4.27: 5’-CCAACAGTACCGGATTGCCAAGCTTGGCCGCCGA-3’. All constructs were verified by DNA sequencing.

For the assay, 1 × 10^5^ cells were seeded in 0.5 mL of growth medium per well in a 24-well plate and incubated for 24 h. The cells were then co-transfected with 500 ng of the *ITGB4* promoter reporter vector and 500 ng of the pGL4.74 [*hRluc*/TK] vector (#E692A, Promega) as an internal control, using 1.5 µL of the *Trans*IT-X2 Dynamic Delivery System (#MIR 6003, Mirus). For the negative control, the pGL4.27 ΔminP vector was used instead of the *ITGB4* promoter reporter vector. At 24 h post-transfection, the medium was replaced with fresh growth medium containing 0 or 40 µM GEM, followed by an additional 48 h of incubation. Luciferase activities were measured using the Dual-Luciferase Reporter Assay System (#E1910, Promega) on a luminometer (GloMax 20/20, #E5311, Promega) according to the manufacturer’s protocol. Firefly luciferase activity was normalized to *Renilla* luciferase activity and expressed as relative luciferase units (RLU).

### In silico expression analysis in the Oncomine, TNMplot and TCGA datasets

The GEO RNA-seq dataset from GREIN (GSE110580) was analyzed using RNAseqChef (https://imeg-ku.shinyapps.io/RNAseqChef/) [16]. Publicly available microarray and RNA-seq datasets from human PDAC patient samples were analyzed using Oncomine (Thermo Fisher Scientific, Badea Pancreas) [17] and TNMplot (https://tnmplot.com/analysis) [18]. The co-expression of the *ITGB4* and laminin-332 subunit genes (*LAMA3*, *LAMB3*, and *LAMC2*) in patients with PDAC was analyzed via RNA-Seq data (TCGA, https://cancergenome.nih.gov; TCGA PanCancer Atlas, *n* = 153) and is shown in graphs generated via cBioPortal (https://www.cbioportal.org).

### Kaplan‒Meier analysis of relapse-free survival

The Kaplan‒Meier analyses of overall survival and relapse-free survival were generated via an online source (http://kmplot.com/analysis).

### Statistics and reproducibility

The results are presented as mean ± SEM and are representative of at least three independent experiments for all *in vitro* studies. Statistical comparisons were performed between two groups using unpaired two-tailed Student’s *t*-test and among the groups using one-way ANOVA followed by Tukey’s post-hoc test with GraphPad Prism Version 6 and SPSS statistics 26 software. A *P*-value of < 0.05 was considered to indicate statistical significance. The Western blot and micrograph results shown are representative images of at least three independent experiments, with similar results.

## Results

### Gemcitabine resistance exacerbates the phenotypes of PDAC cells

**Fig. 1:**
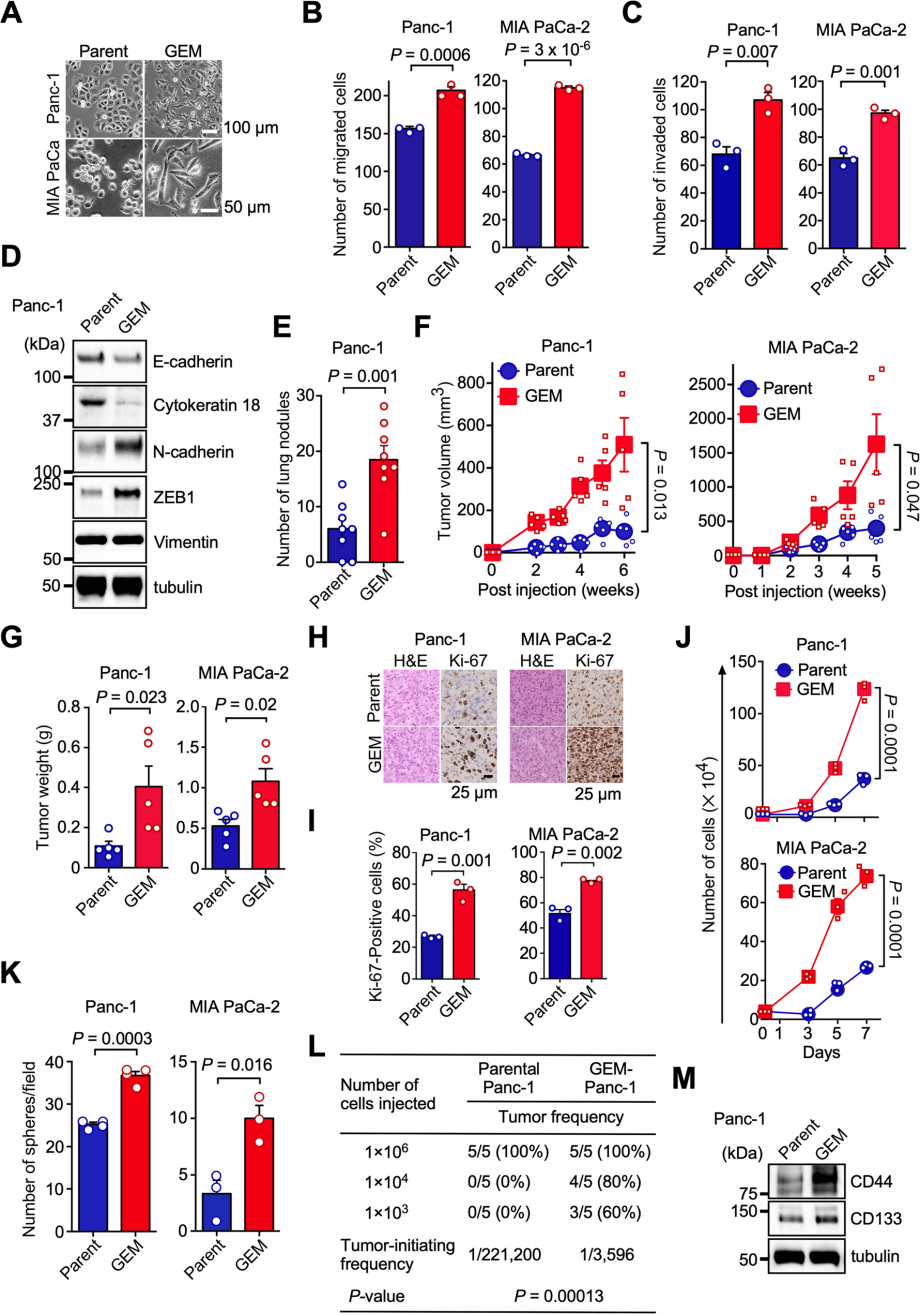
Gemcitabine resistance is associated with an aggressive phenotype in pancreatic ductal adenocarcinoma (PDAC) cells. (**A**) Representative morphology of parental and gemcitabine-resistant (GEM) PDAC cells. (**B**,** C**) Transwell migration (B) and Matrigel invasion (C) assays comparing parental and GEM-PDAC cells. (**D**) Western blot analysis of EMT markers in parental and GEM-resistant Panc-1 cells. Tubulin served as a loading control. (**E**) *In vivo* Lung metastasis assay. Parental or GEM-Panc-1 cells were injected into nude mice via the tail vein. The number of metastatic nodules was quantified 6 weeks post-injection (*n* = 8 per group). (**F**,** G**) *In vivo* subcutaneous xenograft tumor volume (F) and weight (G) of parental and GEM-PDAC cells (*n* = 5 per group). (**H**,** I**) Representative images of H&E and Ki-67 staining (H), and quantification of Ki-67-positive cells (I) in xenograft tumor sections (parental vs. GEM-PDAC cells; *n* = 3 per group). (**J**) *In vitro* proliferation kinetics of parental and GEM-PDAC cells. Cells were seeded at a density of 2.7 × 10^4^ per dish, and cell counts were performed at the indicated time points. (**K**) *In vitro* sphere formation assay of parental and GEM-PDAC cells. (**L**) *In vivo* limiting dilution tumor initiation assay. Parental and GEM-Panc-1 cells were subcutaneously injected into nude mice in a limiting dilution series (1 × 10^6^, 1 × 10^4^, and 1 × 10^3^ cells per injection; *n* = 5 for each dose). The table shows the number of tumor-positive mice and the estimated tumor-initiating frequencies, respectively. Frequencies and *P*-value were calculated using Extreme Limiting Dilution Analysis (ELDA). (**M**) Western blot analysis of stemness markers in parental and GEM-Panc-1 cells. Tubulin served as a loading control. Data in (B, C, E, F, G, I, J, K) are presented as mean ± SEM. For *in vitro* assays (B, C, J, K), data represent at least three independent experiments performed in triplicate. For *in vivo* assays (E–G) and IHC analysis (I), *n* denotes the number of mice per group as indicated. Statistical significance was determined by a two-tailed unpaired Student’s *t*-test

GEM, a standard chemotherapeutic agent for treating PDAC, is often ineffective due to the development of resistance in most patients within weeks of treatment initiation, resulting in a poor prognosis [5, 7, 19]. To investigate the effect of GEM resistance on PDAC progression, two PDAC cell lines, Panc-1 and MIA PaCa-2, were treated with GEM. GEM-resistant Panc-1 (GEM-Panc-1) and MIA PaCa-2 (GEM-MIA PaCa-2) cells were established via exposure to 1.2 µM and 50 nM GEM, respectively. Compared to parental cells, which maintained a clustered and epithelial morphology, GEM-resistant cells exhibited a distinctly scattered, spindle-like mesenchymal-like morphology (Fig. 1A). To test whether GEM resistance enhances malignant potential, we assessed the migratory and invasive capacities of parental and GEM-resistant PDAC cells using Transwell migration and Matrigel invasion assays. Compared with parental cells, GEM-resistant cells presented enhanced invasive phenotypes (Fig. 1B, C). To ensure that the increased number of migrated cells was not a result of accelerated proliferation, we confirmed that parental and GEM-resistant cells exhibited comparable growth rates under the specific low-serum conditions and timeframes used for these assays (Fig. S1). These findings effectively decouple migratory behavior from cell-cycle-driven growth, confirming that GEM resistance directly enhances the invasive phenotype of PDAC cells. The enhanced malignancy was accompanied by a clear transition in epithelial-to-mesenchymal transition (EMT)-related markers (Fig. 1D). Specifically, GEM-Panc-1 cells exhibited a downregulation of E-cadherin and cytokeratin 18, coupled with an upregulation of N-cadherin and ZEB1. Notably, while vimentin levels remained stable due to their high basal expression in the parental line, the induction of ZEB1 and N-cadherin suggests a functional maturation of the partial EMT program, providing a molecular basis for the increased invasiveness. Given that invasive phenotypes are often associated with metastasis, we examined the metastasis of both parental and GEM-Panc-1 cells in a mouse model. Compared with parental cells, GEM-Panc-1 cells resulted in enhanced lung metastasis in nude mice (Fig. 1E). These results suggest that GEM resistance promotes the invasion and metastasis of PDAC cells.

To further investigate the effect of GEM resistance on PDAC progression, we subcutaneously injected parental or GEM-resistant PDAC cells into nude mice and examined primary tumor growth. Mice injected with GEM-resistant cells developed larger tumors than those injected with parental cells (Fig. 1F, G), suggesting that GEM resistance promotes PDAC tumorigenesis. To further explore the underlying mechanism by which GEM-resistant cells exhibit high tumorigenicity, we performed immunohistochemical staining with an anti-Ki-67 antibody on tissue sections from tumors formed by parental cells and GEM-resistant cells. The staining revealed a greater proportion of Ki-67-positive proliferating tumor cells in the GEM-resistant group than in the parental group (Fig. 1H, I). In line with this observation, compared with parental cells, GEM-resistant cells demonstrated increased *in vitro* proliferation (Fig. 1J). We subsequently investigated whether GEM resistance affects the stemness of cells because CSCs are resistant to chemotherapy [20]. Compared with parental cells, GEM-resistant cells exhibited increased *in vitro* sphere formation (Fig. 1K). To further evaluate tumor-initiating capacity *in vivo*, a limiting dilution assay was performed and analyzed using the Extreme Limiting Dilution Analysis (ELDA) software (Fig. 1L, S2). The estimated CSC frequency was 1/3,596 (95% CI: 1/1,309–1/9,879) for GEM-Panc-1 cells versus 1/221,200 (95% CI: 1/41,392–1/1,182,103) for parental Panc-1 cells, representing an approximately 61-fold increase in the GEM-Panc-1 population. The difference was highly significant (*χ*^2^ = 14.6, *P* = 0.00013). The validity of this estimation was supported by the likelihood ratio test for the single-hit model (*P* = 0.298), confirming that the data fit the limiting dilution assumptions. Consistent with these enhanced stem-like properties, the expression levels of CSC markers CD44 and CD133 were markedly upregulated in GEM-Panc-1 cells compared to parental Panc-1 cells (Fig. 1M). These findings show that the development of GEM resistance promotes cell proliferation and stemness in PDAC cells. Taken together, these results suggest that GEM resistance promotes PDAC progression.

### Gemcitabine resistance induces extracellular matrix (ECM)-related gene reprogramming in PDAC cells

**Fig. 2:**
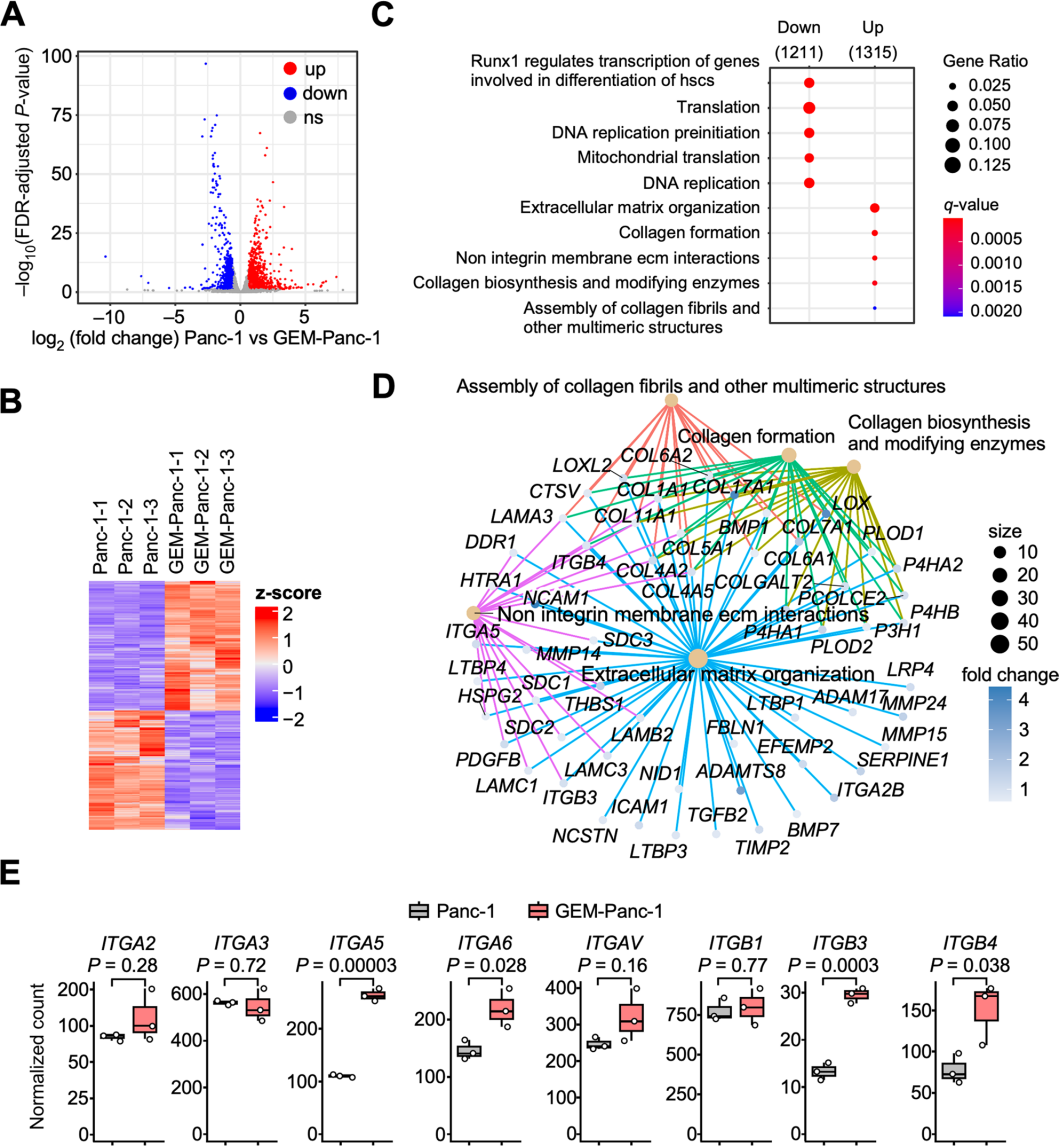
Gemcitabine resistance induces ECM-related gene reprogramming in Panc-1 cells. (**A**) Volcano plot showing differentially expressed genes (DEGs) between parental and GEM-Panc-1 cells. Red and blue dots indicate genes with a > 1.5-fold increase and decrease in expression, respectively (FDR < 0.05, Benjamini‒Hochberg adjusted). The *x*-axis represents log_2_ fold change, and the *y*-axis represents −log_10_ (adjusted *P*-value). (**B**) Heatmap showing 2,526 DEGs identified by RNA-seq in parental and GEM-Panc-1 cells (*n* = 3 per group; FDR < 0.05). In GEM-Panc-1 cells, 1,315 genes were upregulated (red) and 1,211 genes were downregulated (blue). (**C**) Dot plot of Reactome pathway enrichment analysis for DEGs. Bubble size and color represent the gene ratio and *q*-value, respectively. (**D**) Gene-concept network visualizing the top five enriched Reactome pathways. (**E**) Normalized counts of integrin gene expression levels between parental and GEM-Panc-1 cells. Data are presented as mean ± SEM (*n* = 3). Statistical significance was determined by a two-tailed unpaired Student’s *t*-test

To elucidate the mechanisms underlying cancer progression in GEM-resistant cells, we performed gene expression profiling via the GEO RNA-seq dataset from GREIN (GSE110580). A volcano plot and hierarchical cluster analysis demonstrated that, compared with parental Panc-1 cells, GEM-resistant Panc-1 cells presented a distinct expression profile (Fig. 2A, B). Enrichment analysis revealed that the expression of 2,526 genes was markedly altered in GEM-resistant Panc-1 cells compared with parental cells, with notable upregulation of ECM organization-related genes, including collagens, laminins, and integrins (Fig. 2C, D).

Integrin signaling plays a pivotal role in processes related to tumor progression, including cancer cell dissemination, proliferation, and stemness. Consequently, it can be hypothesized that certain integrins may contribute to GEM-induced cancer progression. To investigate this, we analyzed the mRNA expression levels of various integrins in both parental and GEM-Panc-1 cells. As demonstrated in Fig. 2E, GEM resistance led to an increase in the expression of the *ITGA5*, *ITGA6*, *ITGB3*, and *ITGB4* genes, whereas the *ITGA2*, *ITGA3*, *ITGAV*, and *ITGB1* genes remained unresponsive.

### Gemcitabine resistance upregulates β4 integrin expression in PDAC cells

**Fig. 3:**
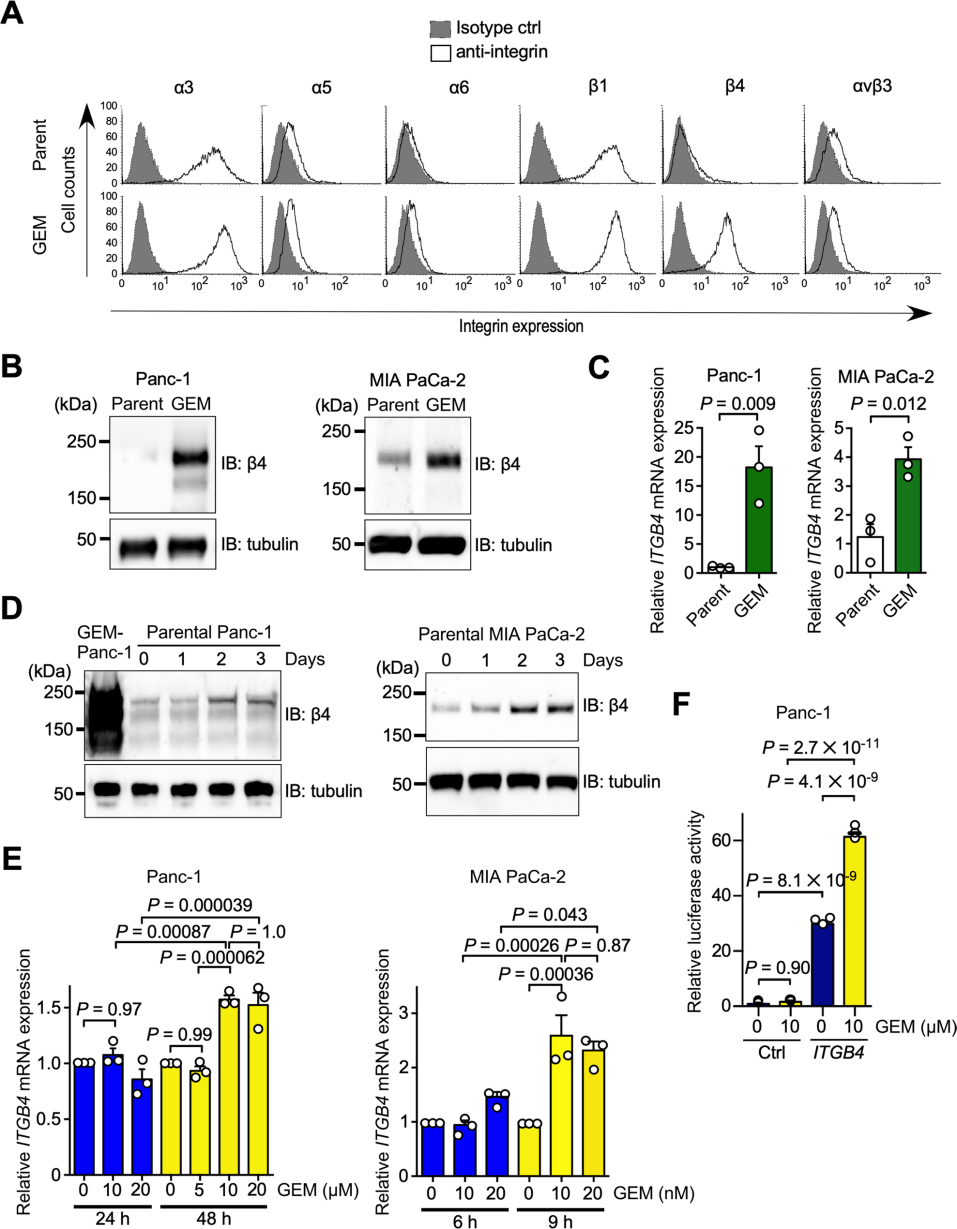
Gemcitabine treatment and the acquisition of drug resistance increase β4 integrin expression in PDAC cells. (**A**) FACS analysis of surface integrin expression in parental and gemcitabine-resistant (GEM) Panc-1 cells. (**B**,** C**) Western blot (B) and RT‒qPCR (C) analysis of β4 integrin *(ITGB4)* expression in parental and GEM-resistant PDAC cells. (**D**) Time-course Western blot analysis of β4 integrin expression in Panc-1 and MIA PaCa-2 cells treated with 20 µM and 20 nM GEM, respectively, for the indicated durations. (**E**) RT‒qPCR analysis showing dose- and time-dependent changes in *ITGB4* mRNA expression following GEM treatment. (**F**) Luciferase reporter assay in Panc-1 cells. Cells were co-transfected with the *ITGB4* promoter reporter and *Renilla* luciferase vectors, treated with GEM 24 h post-transfection, and incubated for an additional 48 h. Results are expressed as relative luciferase units (RLU), normalized to *Renilla* activity. The empty vector (pGL4.27 ΔminP) served as a negative control (Ctrl). Data in (C, E, F) are presented as mean ± SEM from at least three independent experiments performed in triplicate. Statistical significance was determined by a two-tailed unpaired Student’s *t*-test (C) or one-way ANOVA followed by Tukey’s post-hoc test (E, F)

Integrins function as heterodimers on the cell surface; for instance, the α2, α3, α5, α6, αv, β1, β3, and β4 subunits from α2β1, α3β1, α5β1, α6β1, α6β4, αvβ1, and αvβ3 complexes, respectively [21]. Given that *ITGA5*, *ITGA6*, *ITGB3*, and *ITGB4* –but not *ITGA2*, *ITGA3*, *ITGAV* and *ITGB1–* were upregulated in GEM-Panc-1 cells (Fig. 2E), we hypothesized that α6β4 integrin is the primary complex induced during the acquisition of GEM resistance. To test this, we analyzed cell surface expression levels of integrins in parental and GEM-Panc-1 cells via FACS. The levels of the α3, α5, and β1 subunits, as well as the αvβ3 integrin, did not differ between Panc-1 and GEM-Panc1 cells (Fig. 3A). In contrast, the β4 integrin subunit was markedly upregulated, and the α6 integrin subunit was predominantly upregulated in GEM-Panc-1 cells compared with Panc-1 cells (Fig. 3A). This increased β4 integrin expression was also confirmed by Western blot in GEM-Panc-1 and GEM-MIA PaCa-2 cells (Fig. 3B). Consistently, qPCR revealed a significant upregulation of *ITGB4* at the mRNA level in GEM-resistant cells compared to parental cells (Fig. 3C). Time-course Western blot analysis showed a robust increase in β4 integrin protein levels starting two days after GEM treatment (Fig. 3D). To determine whether this induction results from transcriptional regulation, we examined *ITGB4* mRNA expression levels. GEM treatment induced *ITGB4* mRNA in a dose- and time-dependent manner (Fig. 3E). Notably, *ITGB4* mRNA levels markedly increased within 9 h of 10 nM GEM treatment in MIA PaCa-2 cells, suggesting a rapid transcriptional response. Furthermore, a promoter-reporter assay demonstrated that *ITGB4* promoter activity was significantly higher in GEM-Panc-1 cells than in parental Panc-1 cells, and was further enhanced upon acute GEM treatment (Fig. 3F). Collectively, these results suggest that GEM promotes β4 integrin expression through the stimulation of its transcription, leading to elevated protein levels on the cell surface.

### β4 integrin promotes cancer progression in GEM-resistant PDAC cells

**Fig. 4:**
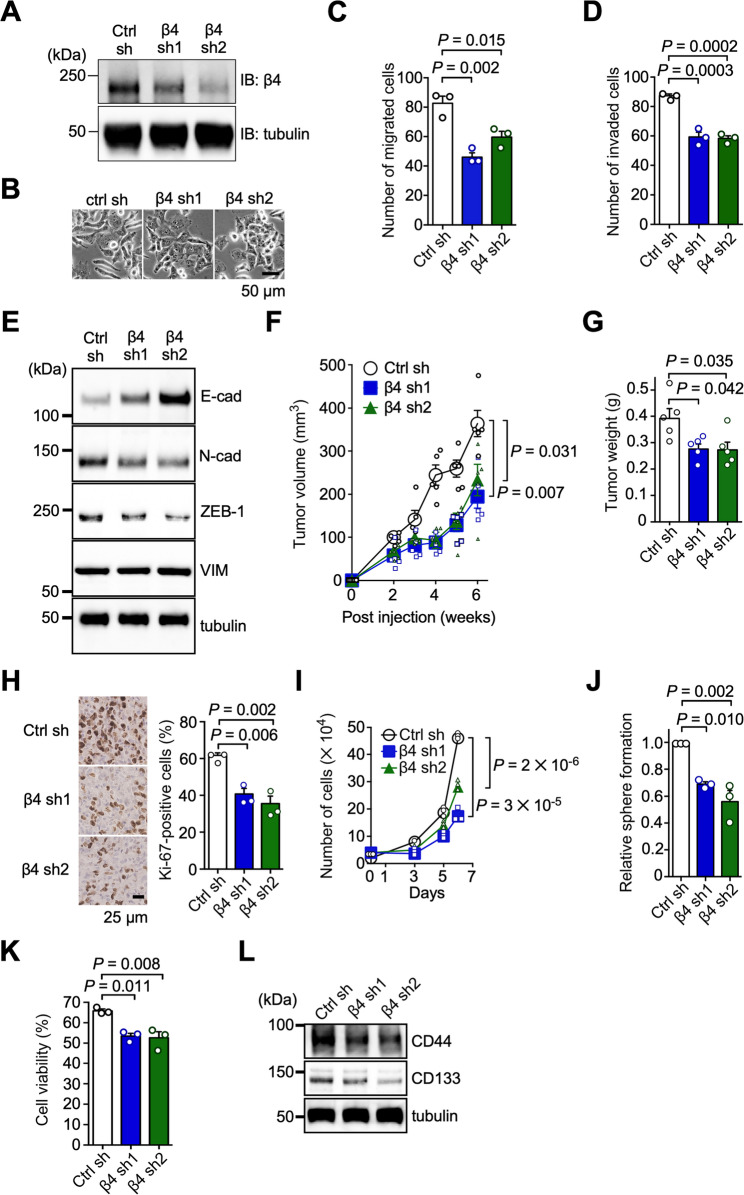
β4 integrin is required for the aggressive phenotype and progression of GEM-Panc-1 cells. (**A**) Western blot analysis of β4 integrin in GEM-Panc-1 cells expressing control shRNA (Ctrl sh) or two independent β4 integrin-specific shRNAs (β4 sh1 and β4 sh2). (**B**) Representative morphology of the indicated GEM-Panc-1 cells. (**C**,** D**) Transwell migration (C) and Matrigel invasion (D) assays of GEM-Panc-1 cells expressing Ctrl sh, β4 sh1, or β4 sh2. (**E**) Western blot analysis of EMT markers in the indicated cells. Tubulin served as a loading control. (**F**,** G**) Xenograft tumor volume (F) and weight (G) in nude mice subcutaneously injected with GEM-Panc-1 cells expressing Ctrl sh, β4 sh1, or β4 sh2 (*n* = 5 per group). (**H**) Representative images of Ki-67 immunohistochemistry staining in xenograft sections (left) and quantification of Ki-67-positive cells (right) (*n* = 3 per group). (**I–K**) *In vitro* cell proliferation (I), sphere formation (J), and viability (K) assays of GEM-Panc-1 cells expressing Ctrl sh, β4 sh1, or β4 sh2. For the proliferation assay, cells were seeded at a density of 2 × 10^4^ cells/dish, and cell counts were performed at the indicated time points. For the viability assay, cells were treated with 4 mM GEM for 72 h; viability was normalized to the respective untreated controls. **(L)** Western blot analysis of stemness markers in the indicated GEM-Panc-1 cells. Data in (C, D, F, G, H, I, J, K) are presented as mean ± SEM. For *in vitro* assays (C, D, I, J, K), data represent at least three independent experiments performed in triplicate. For *in vivo* assays (F, G) and immunohistochemical analysis (H), *n* denotes the number of mice per group. Statistical significance was determined by one-way ANOVA followed by Tukey’s post-hoc test

Although β4 integrin is a structural component of the hemidesmosomal α6β4 integrin complex, it is highly upregulated in PADC tumors [22, 23]. In cancer cells, β4 integrin promotes proliferation, migration, and stemness, thereby driving tumorigenesis and metastasis [23, 24]. Given that GEM-resistant cells exhibit high β4 integrin expression, we hypothesized its involvement in the malignancy of these cells. To test this, we established two stable β4 integrin-knockdown GEM-Panc-1 cell lines (β4 sh1 and β4 sh2) along with a stable control shRNA (ctrl sh) line (Fig. 4A). shRNA-mediated β4 integrin knockdown suppressed cell elongation and scattering in GEM-Panc-1 cells (Fig. 4B). We further examined the impact of this knockdown on cancer progression. Compared with the control, the knockdown of β4 integrin markedly decreased GEM-Panc-1 cell migration and invasion (Fig. 4C, D). This reduced malignancy was accompanied by the upregulation of E-cadherin and downregulation of N-cadherin/ZEB1, indicating a partial mesenchymal-to-epithelial transition (MET), as vimentin levels remained unchanged (Fig. 4E). In a mouse tumor xenograft model, β4 integrin-knockdown GEM-Panc-1 cells (β4 sh1 and β4 sh2) exhibited reduced tumor volume (Fig. 4F) and weight (Fig. 4G) compared to ctrl sh cells, suggesting that β4 integrin expression is essential for tumorigenicity. To elucidate the underlying mechanism, we performed Ki-67 staining on tumor sections. Consistent with the reduced tumor growth, β4 sh GEM-Panc-1 tumors presented fewer Ki-67-positive cells than the controls (Fig. 4H). Furthermore, *in vitro* cell proliferation (Fig. 4I) and sphere formation (Fig. 4J) assays revealed that β4 integrin knockdown decreased both cell growth and stemness. Notably, both β4 sh1 and sh2 GEM-Panc-1 cells showed reduced viability in response to GEM (Fig. 4K). This loss of stem-like properties was further supported by the substantial downregulation of CSC markers CD44 and CD133 (Fig. 4L). Collectively, our findings suggest that β4 integrin promotes the motility, tumorigenicity, chemoresistance of GEM-resistant PDAC cells by enhancing cell proliferation and stemness.

### β4 integrin overexpression is associated with poor prognosis in PDAC patients

**Fig. 5:**
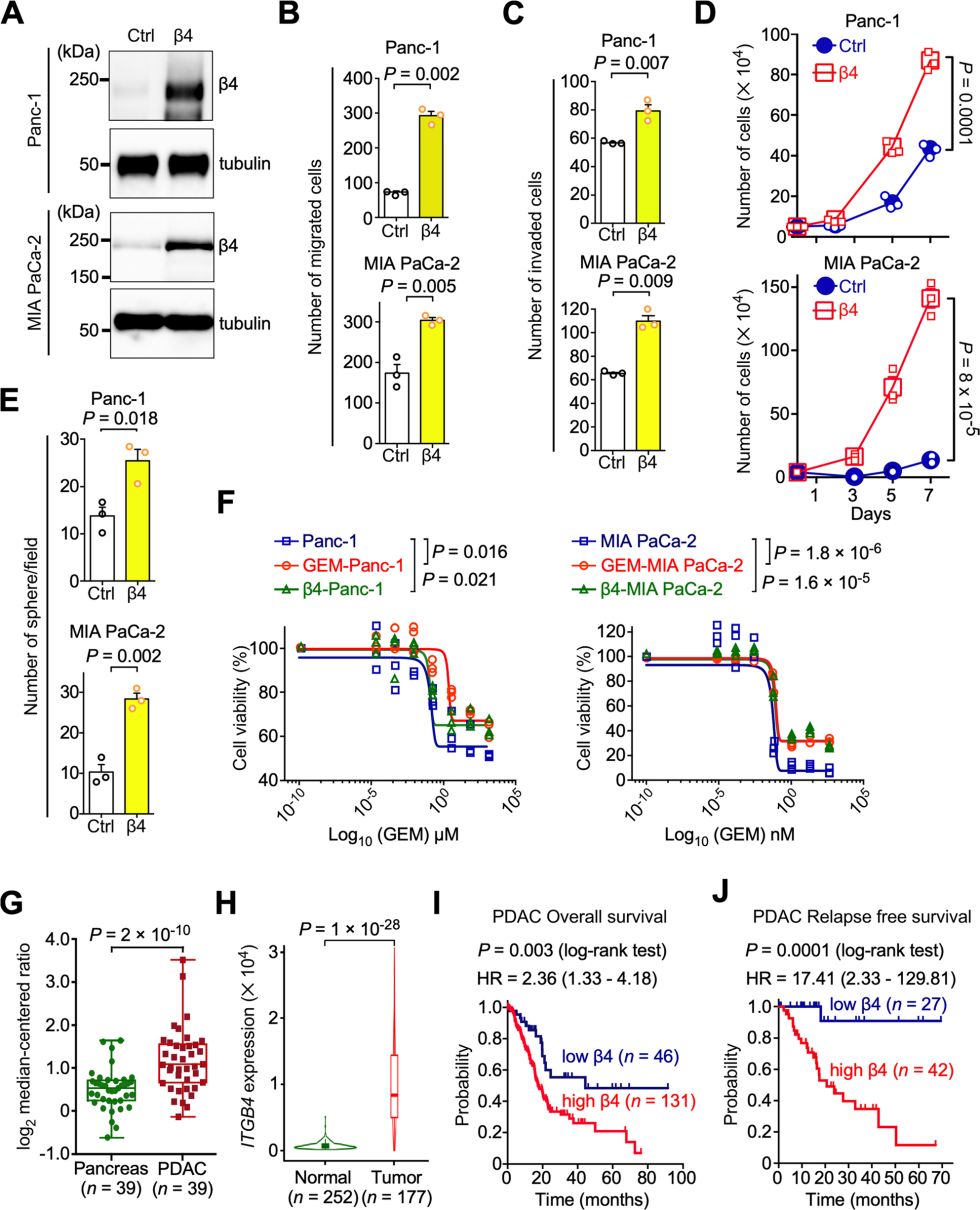
β4 integrin expression is associated with PDAC progression and poor patient prognosis. **A** Western blot analysis of β4 integrin expression in Panc-1 and MIA PaCa-2 cells stably overexpressing β4 integrin (β4) or a control vector (Ctrl). **B–E** Effects of β4 integrin overexpression on cell migration (B), invasion (C), proliferation (D), and sphere formation (E). For the cell proliferation assay, 5 × 10^4^ cells of Panc-1 or 4 × 10^4^ cells of MIA PaCa-2 were seeded per dish, and cell counts were performed at the indicated time points. **F** Cell viability assay of parental, GEM-resistant, and β4-overexpressing cells following GEM treatment for 72 h. The bottom plateaus were 55.38% (95% CI: 49.79 to 60.97) for Panc-1, 67.11% (95% CI: 62.5 to 71.72) for GEM-Panc-1, and 65.04% (95% CI: 61.97 to 68.12) for B4-Panc-1 cells. For the MIA PaCa-2 series, bottom plateaus were 7.615% (95% CI: -3.979 to 19.21) for MIA PaCa-2, 31.55% (95% CI: 29.19 to 33.90) for GEM-MIA PaCa-2, and 31.83% (95% CI: 27.96 to 35.7) for ß4-MIA PaCa-2 cells.. Curves were fitted using a four-parameter logistic model with the top plateau constrained to 100%, while the bottom plateau was allowed to float to account for the partial response observed in resistant lines (goodness-of-fit: *R*^2^ > 0.86 for all curves). For MIA PaCa-2 cell lines, the Hill slope was constrained to − 20, while no constraints were applied to Panc-1 cell lines. *P-*values for the comparison of bottom plateaus were determined by one-way ANOVA followed by Tukey’s post-hoc test. *IC*_50_ values and 95% CIs are provided in the Results section. **G**,** H** *ITGB4* mRNA expression in human normal pancreas and PDAC tumor tissues from publicly available datasets ([17] for G and [18] for H). **I**,** J** Kaplan‒Meier analysis of the correlation between *ITGB4* mRNA expression and overall survival (I) or relapse-free survival (J) in PDAC patients using the Kaplan‒Meier Plotter database. Data in (B–F) represent mean ± SEM from at least three independent experiments performed in triplicate. Data in (G, H) are presented as mean ± SEM from the indicated number of patient samples (*n*). Statistical significance was determined by a two-tailed unpaired Student’s *t*-test (B–E, G, H) or the log-rank test (I, J)

The malignant potential of GEM-Panc-1 cells was abrogated by decreased expression of β4 integrin in these cells. To obtain direct evidence that β4 integrin expression enhances the malignancy of PDAC cells, we sought to generate β4 integrin-overexpressing PDAC cell lines. To generate stable β4 integrin-PDAC transfectants, Panc-1 and MIA PaCa-2 cells were retrovirally transduced with the *ITGB4* (Fig. 5A, β4-Panc-1 and β4-MIA PaCa-2 cells). To determine the effect of β4 integrin expression on PDAC progression, we examined the migration, invasion, proliferation, and sphere formation of control and β4 integrin-overexpressing cells. β4 integrin overexpression markedly enhanced cell migration (Fig. 5B) and Matrigel invasion (Fig. 5C) in both PDAC cell lines. Furthermore, β4 integrin-overexpressing PDAC cells exhibited notably greater proliferative and stemness potential than control cells (Fig. 5D, E). In addition, nonlinear regression analysis of the Panc-1 series revealed that β4 integrin expression shifted the survival limit from 55% to 65% without altering the *IC*_50_ threshold (~ 1.3 µM), whereas the long-term selected GEM-Panc-1 cells showed a marked *IC*_50_ shift to 177.1 µM (Fig. 5F). Similarly, in the MIA PaCa-2 series, the *IC*_50_ values for MIA PaCa-2, GEM-MIA PaCa-2, β4-MIA PaCa-2 were 1.134 nM (95% CI: 1.089–1.182), 1.213 nM (1.195–1.231), and 1.183 nM (1.159 − 1.207), respectively, showing no evident shift in drug potency. In contrast, a profound shift was observed in the maximal therapeutic efficacy (bottom plateau) in these cells (Fig. 5F). While parental MIA PaCa-2 cells were nearly eliminated (~ 10% survival), β4-MIA PaCa-2 cells exhibited a substantially higher survival limit of ~ 32% (95% CI: 27.96–35.70%), which was comparable to that of the GEM-MIA PaCa-2 cells (95% CI: 29.19–33.90%). These results demonstrate that β4 integrin primarily contributes to GEM resistance by limiting the maximal extent of cell death (reducing efficacy) rather than shifting the initial drug sensitivity threshold (potency). Taken together, these findings suggest that β4 integrin plays a pivotal role in the progression and chemoresistance of PDAC.

To evaluate the clinical association between β4 integrin expression and poor outcomes in patients with PDAC, we analyzed two publicly available datasets of human patient samples. The Baeda Pancreas microarray dataset from the Oncomine database revealed a significant increase in *ITGB4* mRNA expression in PDAC tissues (*n* = 39) compared with matched normal pancreatic tissues (*n* = 39) (Fig. 5G). Another RNA-seq dataset from the TNMplot database also revealed a relatively high level of *ITGB4* mRNA expression in PDAC tissues (*n* = 177) compared with normal pancreatic tissues (*n* = 252) (Fig. 5H). Clinically, Kaplan‒Meier analysis revealed that high *ITGB4* mRNA expression was associated with poor overall survival (Fig. 5I) and relapse-free survival (Fig. 5J), suggesting that high *ITGB4* expression is associated with poor prognosis in patients with PDAC.

### The β4 integrin/laminin-332 axis promotes cancer progression in GEM-resistant PDAC cells

**Fig. 6:**
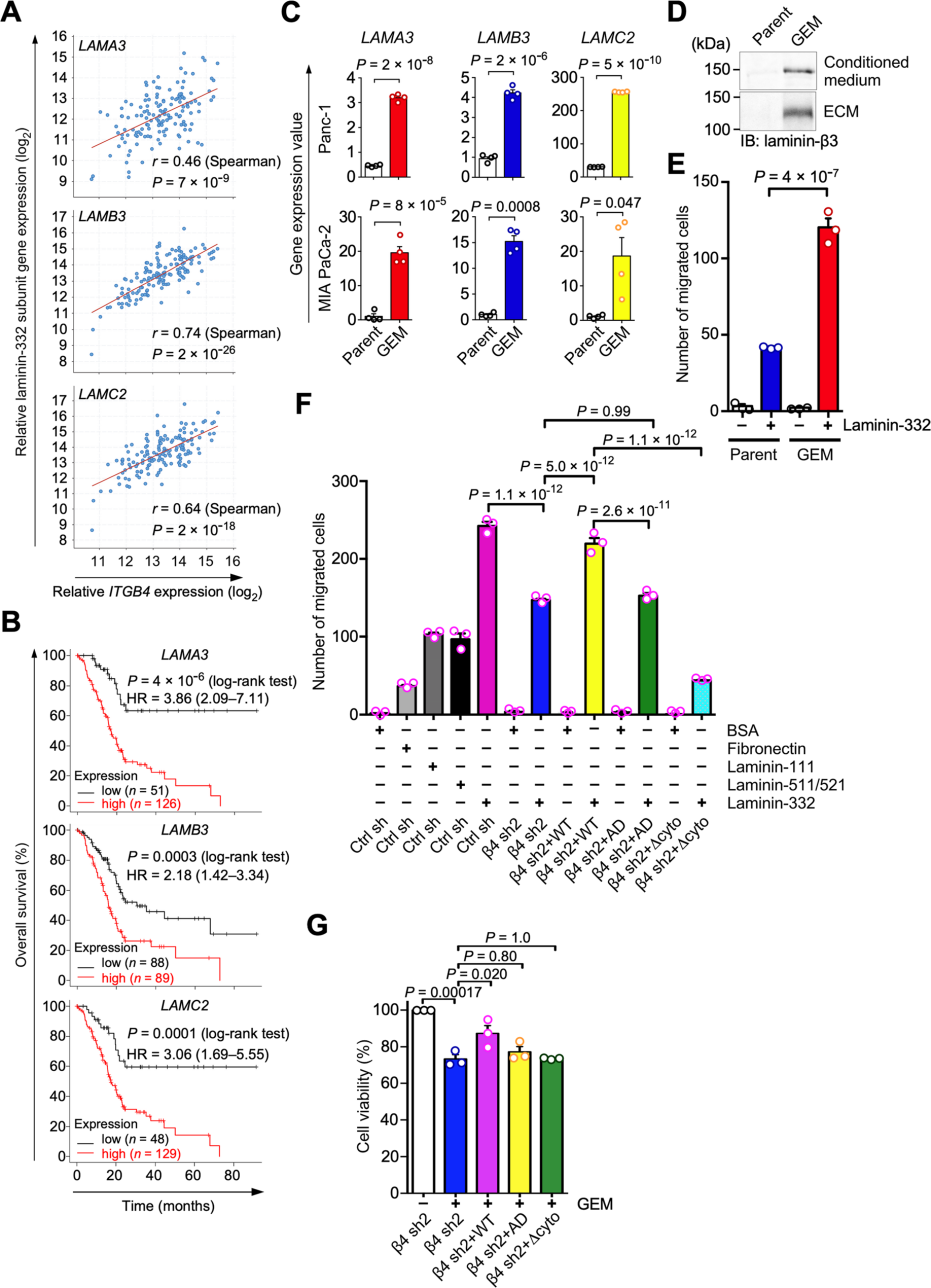
Laminin-332 is associated with poor prognosis and promotes malignancy in GEM-resistant PDAC cells. (**A**) Correlation between mRNA expression of *ITGB4* and laminin-332 subunits (*LAMA3*, *LAMB3*, and *LAMC2*) in PDAC tissues from the TCGA dataset (*n* = 153). Spearman’s rank correlation coefficient (*r*) and *P*-values are shown. (**B**) Kaplan–Meier survival analysis of PDAC patients (*n* = 177) grouped by laminin-332 subunit mRNA levels using the Kaplan–Meier Plotter database. *P*-values were determined by the log-rank test. (**C**) mRNA expression of laminin-332 subunits in parental vs. GEM-treated (GEM) Panc-1 cells (30 nM for 10 days; GSE249302) and MIA PaCa-2 cells (300 nM for 24 h; GSE105083). (**D**) Western blot analysis of secreted and deposited laminin-332 in Panc-1 and GEM-Panc-1 cells. Equal volumes of the conditioned medium and ECM fractions from Panc-1 and GEM-Panc-1 cells were loaded. Although GEM-Panc-1 cells were approximately 1.4-fold fewer than Panc-1 cells at harvest, they exhibited higher levels of laminin-332, suggesting increased production per cell. (**E**) Migration of parental and GEM-Panc-1 cells on 1 µg/mL laminin-332. (**F**) Migration of the indicated GEM-Panc-1 cells on BSA (12 mg/mL), fibronectin (2 µg/mL), laminin-111 (20 µg/mL), laminin-511/521 (2.5 µg/mL), or laminin-332 (2 µg/mL). (**G**) Viability of the indicated GEM-Panc-1 cells treated with 2 mM GEM. Viability was normalized to the respective untreated controls, with the value for untreated β4 sh2-GEM-Panc-1 cells set to 100%. WT, wild-type β4 integrin; AD, adhesion-deficient β4 integrin; Δcyto, cytoplasmic domain-deleted β4 integrin. Data are presented as mean ± SEM from four independent experiments (C) or at least three independent experiments performed in triplicate (E**–**G). Statistical significance was determined by a two-tailed unpaired Student’s *t*-test (C) or one-way ANOVA followed by Tukey’s post-hoc test (E**–**G)

Laminin-332, a heterotrimer consisting of α3 (encoded by *LAMA3*), β3 (encoded by *LAMB3*), and γ2 (encoded by *LAMC2*) subunits, is highly expressed in pancreatic cancer tissues [25] and serves as a primary ligand for β4 integrin. Given that the association between laminin-332 and β4 integrin promotes cancer progression [24], we analyzed the correlation between *ITGB4* expression and the levels of laminin-332 subunit genes in PDAC patients using TCGA RNA-seq data. Our analysis revealed a significant positive correlation between *ITGB4* and the genes encoding laminin-332 subunits (Fig. 6A). Furthermore, Kaplan‒Meier analysis showed that elevated expression of these subunit genes was associated with decreased overall survival in PDAC patients (Fig. 6B).

Next, we investigated the impact of GEM-resistance on the expression of laminin-332 subunit genes in PDAC cells. Analysis of GEO RNA-seq datasets (GSE249302 for Panc-1 and GSE105083 for MIA PaCa-2) revealed that GEM-resistant cells markedly upregulated these genes compared to their parental cells (Fig. 6C). Consistently, Western blot analysis confirmed a striking increase in secreted and deposited laminin-332 in GEM-Panc-1 cells compared to parental Panc-1 cells, where it was barely detectable (Fig. 6D). To compare these levels across conditions, equal volumes of the conditioned medium and ECM fractions were loaded. Notably, this increase was observed despite the fact that the total number of GEM-Panc-1 cells at the time of collection was approximately 1.4-fold lower than that of parental Panc-1 cells. These results suggest the enhanced presence of laminin-332 reflects a genuine upregulation of its synthesis and extracellular accumulation on a per-cell basis, rather than a difference in cell density.

When plated on laminin-332, GEM-Panc-1 cells exhibited substantially higher motility than parental Panc-1 cells (Fig. 6E). In contrast, this GEM resistance-induced enhancement of motility was not observed on other substrates, such as fibronectin, laminin-111, and laminin-511/521, where GEM-Panc-1 cells showed only minimal motility (Fig. 6F). These findings indicate that the increased cell migration is highly substrate-specific, suggesting that the β4 integrin/laminin-332 interaction plays a pivotal role in GEM-induced cancer progression. We therefore investigated whether this specific association is indispensable for the observed enhancement in motility. To this end, we utilized β4 sh2 GEM-Panc-1 cells stably expressing one of two β4 integrin mutants: an adhesion-deficient mutant (ADβ4) lacking laminin-332 binding, or a cytoplasmic domain-deleted mutant (Δcytoβ4) incapable of mediating downstream signaling (Fig. S3). Knockdown of β4 integrin markedly reduced GEM-Panc-1 cell motility on laminin-332 compared to control shRNA (Fig. 6F, Ctrl sh vs. β4 sh2 on laminin-332). This reduction was substantially rescued by the re-expression of wild-type β4 integrin (WTβ4) (Fig. 6F, β4 sh2 vs. β4 sh2+WTβ4 on laminin-332), whereas introduction of the ADβ4 mutant failed to restore motility (Fig. 6F, β4 sh2 vs. β4 sh2+ADβ4 on laminin-332). Notably, Δcytoβ4 expression reduced motility even more than ADβ4 (Fig. 6F, β4 sh2 vs. β4 sh2 + Δcytoβ4 vs. β4 sh2+ADβ4 on laminin-332), suggesting that laminin-332-independent β4 integrin signaling also contributes to GEM-Panc-1 cell migration.

Regarding GEM-resistance, β4 sh2 cells exhibited substantially decreased viability upon GEM treatment (Fig. 6G). While WTβ4 expression successfully rescued GEM resistance, neither ADβ4 nor Δcytoβ4 could do so (Fig. 6G). Collectively, these findings suggest that the interaction between β4 integrin and laminin-332 is essential for both the motility and GEM resistance of GEM-Panc-1 cells, identifying this axis as a potential driver of GEM-induced PDAC progression.

### β4 integrin-dependent Src activation promotes cell motility and GEM resistance in GEM-resistant PDAC cells

**Fig. 7:**
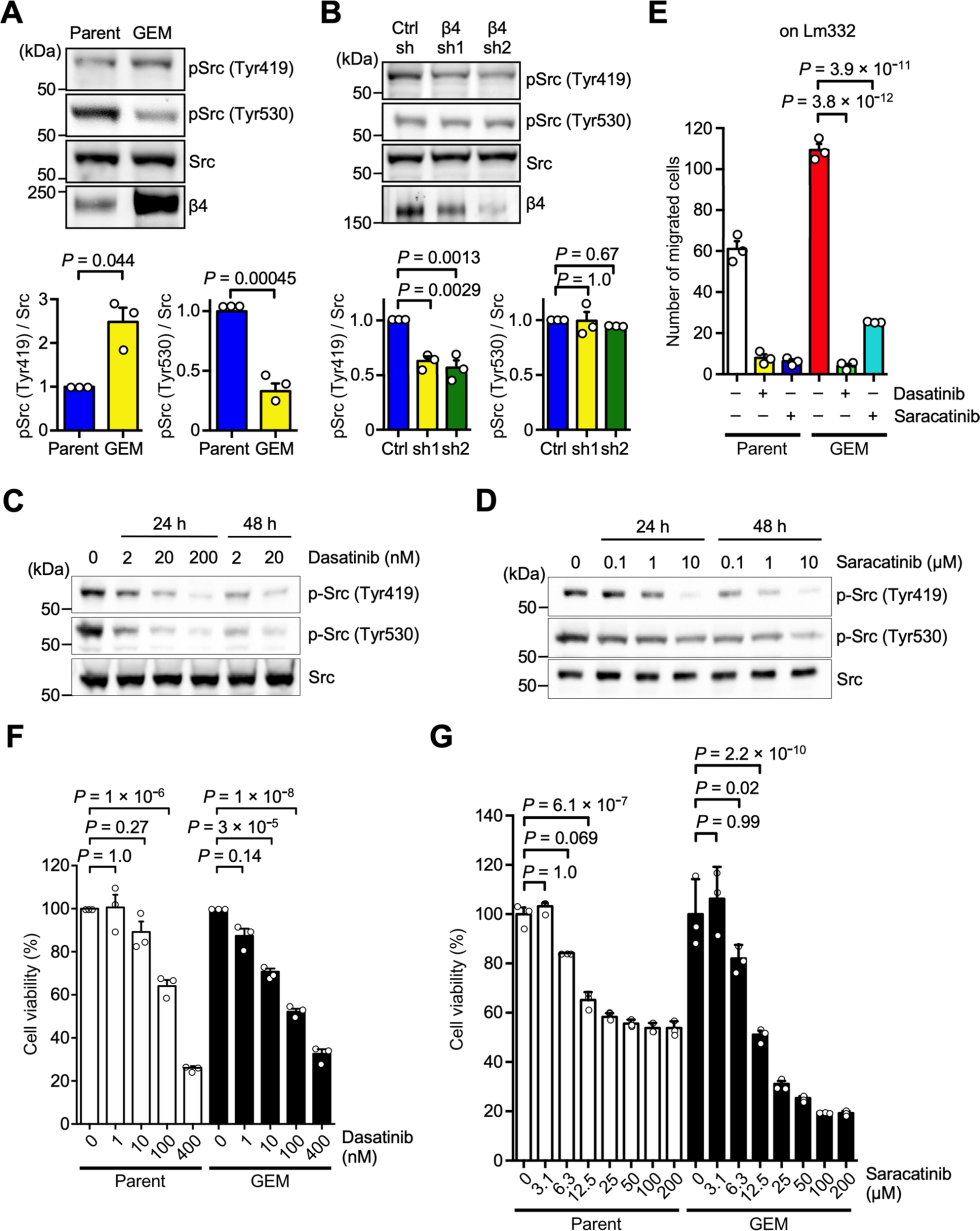
β4 integrin upregulation is associated with activation of the Src signaling pathway in GEM-resistant cells. (**A**) Western blot analysis of Src phosphorylation in parental and GEM-Panc-1 cells. The relative levels of phosphorylated Src were quantified, with the mean value for parental Panc-1 cells set to 1. (**B**) Western blot analysis of Src phosphorylation in GEM-Panc-1 cells expressing control (Ctrl) shRNA or two distinct β4 integrin-specific shRNAs. The relative level for Ctrl shRNA cells was set to 1. (**C**,** D**) Inhibitory effects of Src inhibitors, dasatinib (C) and saracatinib (D), on Src phosphorylation in GEM-Panc-1 cells. DMSO was used as a vehicle control. (**E**) Effects of dasatinib (200 nM) or saracatinib (1 µM) on the migration of parental and GEM-Panc-1 cells on laminin-332 (2 µg/mL). (**F**,** G**) Viability of parental and GEM-Panc-1 cells treated with 500 µM GEM and the indicated concentrations of dasatinib (F) or saracatinib (G). Viability was normalized to cells treated with GEM alone (set to 100%). Data in (A, B, E–G) are presented as mean ± SEM from at least three independent experiments performed in triplicate. Statistical significance was determined by a two-tailed unpaired Student’s *t*-test (A) or one-way ANOVA followed by Tukey’s post-hoc test (B, E– G)

Next, we investigated the mechanisms by which β4 integrin promotes progression in GEM-resistant PDAC cells. Given that β4 integrin is known to activate Src signaling and drive invasive phenotypes [24], we hypothesized that GEM-induced β4 integrin expression promotes cancer progression via Src activation. Western blot analysis revealed that in GEM-Panc-1 cells, Src phosphorylation was elevated at the activating site (Tyr419) but diminished at the inhibitory site (Tyr530) compared to parental cells (Fig. 7A). Notably, β4 integrin knockdown in two stable GEM-Panc-1 cell lines attenuated Src phosphorylation at Tyr419 without affecting Tyr530 levels (Fig. 7B). These results suggest that the upregulation of β4 integrin in GEM-Panc-1 cells selectively enhances Src activation.

Furthermore, we examined whether this β4 integrin-mediated Src activation is essential for the malignant phenotype of GEM-resistant PDAC cells. For this purpose, we employed the potent Src inhibitors dasatinib (Fig. 7C) and saracatinib (Fig. 7D). As expected, both inhibitors markedly suppressed the migration of GEM-Panc-1 cells on laminin-332 (Fig. 7E). Similarly, treatment with dasatinib (Fig. 7F) or saracatinib (Fig. 7G) effectively sensitized GEM-Panc-1 cells to GEM. Notably, the inhibitory effect of these Src inhibitors on GEM resistance was more pronounced in GEM-Panc-1 cells than in parental Panc-1 cells. Taken together, these findings suggest that β4 integrin drives cancer progression in GEM-resistant cells by inducing Src activation.

### β-catenin/p300-mediated histone acetylation upregulates β4 integrin expression in GEM-resistant PDAC cells

**Fig. 8:**
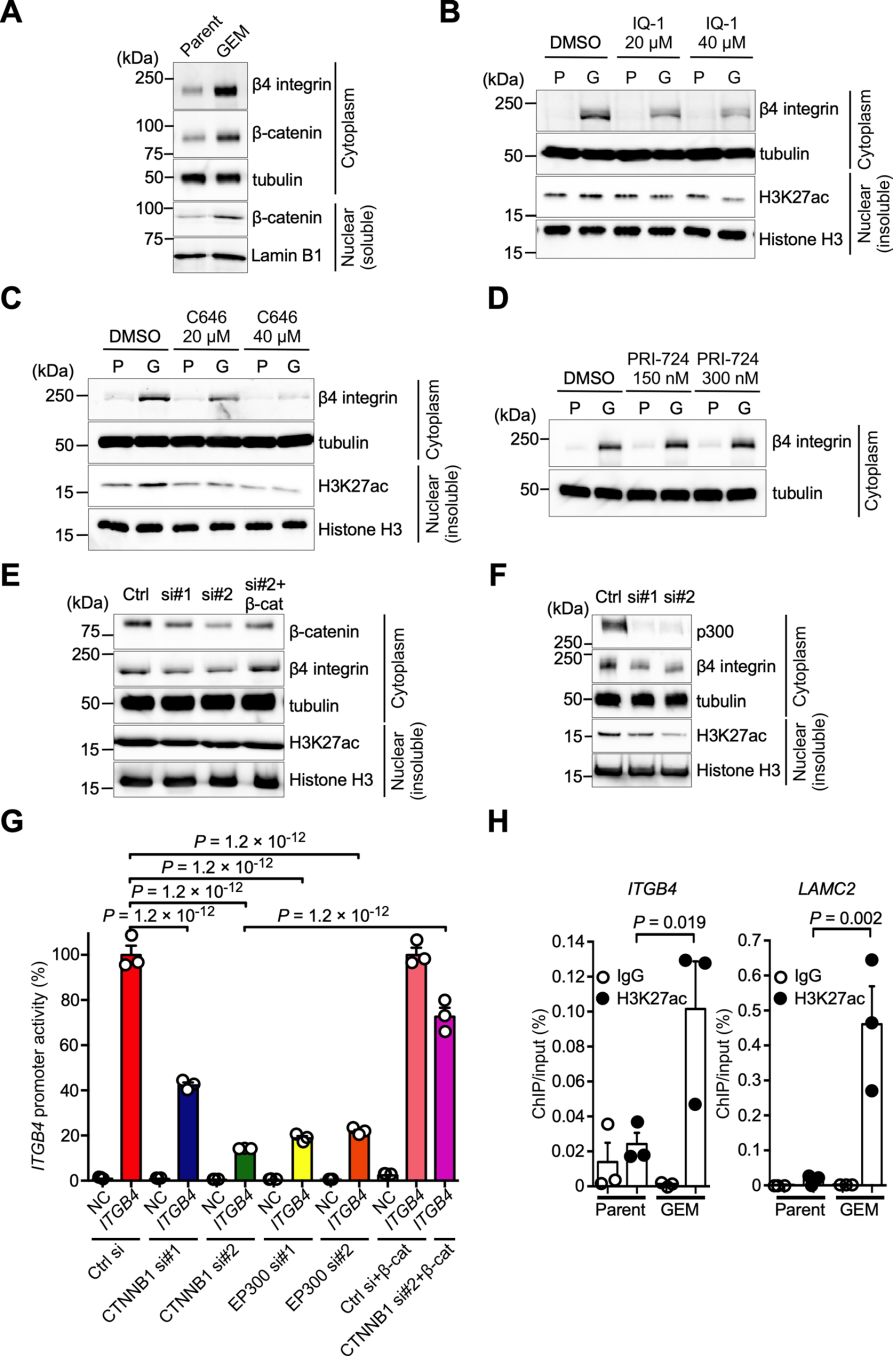
β-catenin-mediated histone acetylation at the *ITGB4* promoter supports β4 integrin expression. **A** Western blot analysis of cytoplasmic and nuclear β-catenin levels in parental and GEM-Panc-1 cells. **B** Effects of IQ-1, an inhibitor of the β-catenin/p300 interaction, on β4 integrin expression and H3K27 acetylation (H3K27ac) levels in parental (P) and GEM (G)-Panc-1 cells. **C** Effects of the p300 inhibitor C646 on β4 integrin expression and H3K27ac levels in parental (P) and GEM (G)-Panc-1 cells. **D** Effect of PRI-724, an inhibitor of the β-catenin/CBP interaction, on β4 integrin expression in parental (P) and GEM (G)-Panc-1 cells. DMSO was used as a vehicle control. **E** Western blot analysis of β4 integrin and H3K27ac in GEM-Panc-1 cells following β-catenin knockdown (CTNNB1 si#1 and #2) or β-catenin rescue (si#2 with β-catenin overexpression). **F** Effect of p300 knockdown (EP300 si#1 and #2) on β4 integrin expression and H3K27ac levels in GEM-Panc-1 cells. **G** Luciferase reporter assay in GEM-Panc-1 and β-catenin-overexpressing GEM-Panc-1 (+β-cat) cells. Cells were co-transfected with the *ITGB4* promoter reporter, *Renilla* luciferase vector, and the indicated siRNAs, followed by incubation for 72 h. Data were normalized to *Renilla* luciferase activity and are presented as a percentage of the control siRNA (Ctrl si), which was set to 100%. The pGL4.27 ΔminP empty vector served as a negative control (NC) for promoter activity. **H** Chromatin immunoprecipitation (ChIP)–qPCR analysis of H3K27ac at the *ITGB4* (β4 integrin) and *LAMC2* (laminin γ2) promoters in parental and GEM-Panc-1 cells. Normal rabbit IgG served as a negative control. Data are expressed as a percentage of the input. Data in (G, H) are presented as mean ± SEM from at least three independent experiments. Statistical significance was determined by one-way ANOVA followed by Tukey’s post-hoc test

Finally, we investigated the mechanism underlying the increased β4 integrin expression in GEM-Panc-1 cells. Elevated levels of cytoplasmic and nuclear β-catenin (encoded by *CTNNB1*) are frequently observed in PDAC and correlate with PanIN grade and the development of invasive disease [26]. Nuclear β-catenin binds to the TCF/LEF family of transcription factors to activate the transcription of target genes [27]. Thus, we hypothesized that Wnt/β-catenin signaling upregulates β4 integrin expression in GEM-Panc-1 cells. To test this, we first examined β-catenin expression in parental and GEM-Panc-1 cells. GEM resistance increased both β-catenin and β4 integrin levels in the cytoplasmic fraction of Panc-1 cells (Fig. 8A). Notably, the nuclear fraction of GEM-Panc-1 cells contained higher levels of β-catenin than that of parental cells (Fig. 8A), indicating enhanced nuclear localization of β-catenin in GEM-resistant cells.

We then examined whether β-catenin/p300 or β-catenin/CBP complexes are involved in enhancing β4 integrin expression. Since nuclear β-catenin and its co-activators, p300 (encoded by *EP300*) or CBP, induce histone H3 lysine 27 acetylation (H3K27ac) at target gene promoters [28], we utilized IQ-1 (a specific inhibitor of the β-catenin/p300 interaction [29]) and PRI-724 (an inhibitor of the β-catenin/CBP interaction [30]). IQ-1 suppressed β4 integrin expression and decreased global H3K27ac levels in GEM-Panc-1 cells (Fig. 8B). This was further substantiated using C646, another p300 inhibitor, which also reduced both β4 integrin and H3K27ac levels (Fig. 8C). Additionally, IQ-1 and C646 substantially inhibited GEM-induced β4 integrin expression in both Panc-1 and MIA PaCa-2 cells (Fig. S4). In contrast, the CBP-specific inhibitor PRI-724 had no discernible effect on β4 integrin expression (Fig. 8D).

To further validate these findings, we performed siRNA-mediated knockdown of β-catenin or p300. Western blot analysis revealed that silencing either β-catenin (Fig. 8E) or p300 (Fig. 8F) reduced β4 integrin protein levels, consistent with the effects of IQ-1 and C646. Supporting this, knockdown of β-catenin or p300 substantially suppressed *ITGB4* promoter-reporter activity in GEM-Panc-1 cells (Fig. 8G). Notably, while p300 depletion reduced H3K27ac levels, β-catenin knockdown did not alter global H3K27ac status, suggesting that β-catenin may regulate *ITGB4* transcription by recruiting p300 to specific genomic loci rather than modulating its overall enzymatic activity. Furthermore, ectopic expression of β-catenin successfully rescued both β4 integrin protein levels (Fig. 8E) and *ITGB4* promoter activity (Fig. 8G) in β-catenin-silenced cells. In contrast, similar rescue experiments with p300 were technically limited, as its overexpression induced significant cytotoxicity, preventing further functional assessment.

Although β-catenin typically activates transcription via TCF/LEF-binding motifs [27], our sequence analysis of the *ITGB4* promoter failed to identify consensus sequences. However, interrogation of the ChIP-Atlas database revealed significant enrichment of β-catenin (*CTNNB1*), p300 (*EP300*), and TCF family members (TCF3, TCF7, and TCF7L2) within the same regulatory window of *ITGB4* promoter (Fig. S5). Notably, these factors co-localized within a region characterized by high levels of H3K27ac, a hallmark of active promoters and enhancers. Although the peak of β-catenin was located slightly upstream of the TCF clusters, the broad overlap of these transcriptional regulators and the histone acetylation marks suggest the formation of a functional transcriptional complex to regulate β4 integrin expression. Consistent with this, our reporter assays suggest a model in which both β-catenin and p300 contribute to *ITGB4* promoter activity (Fig. 8G).

The *ITGB4* promoter region was characterized by high levels of H3K27ac (Fig. S5). ChIP‒qPCR analysis demonstrated that the *ITGB4* promoter is markedly more acetylated in GEM-resistant cells than in parental cells (Fig. 8H). Similar results were observed for the *LAMC2* gene (Fig. 8H), suggesting a common epigenetic regulatory mechanism. Collectively, these findings support a model in which the β-catenin/p300 complex contributes to *ITGB4* transcription, associated with H3K27ac deposition. This mechanism, potentially involving non-canonical sequences or indirect recruitment, underscores the epigenetic reprogramming that correlates with β4 integrin overexpression and subsequent PDAC progression.

## Discussion

**Fig. 9:**
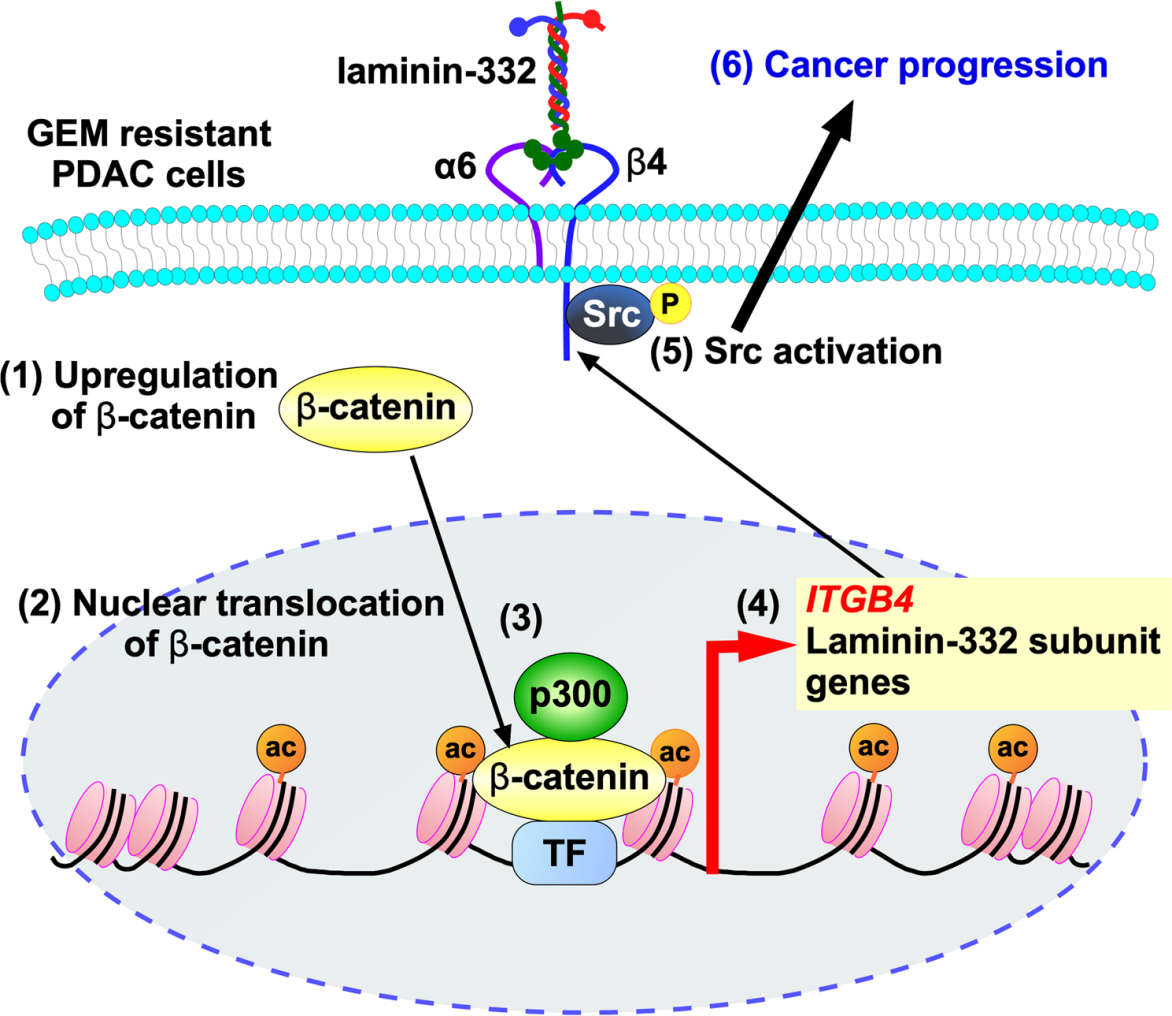
Schematic model of α6β4 integrin-mediated progression in GEM-resistant PDAC cells. In GEM-resistant PDAC cells, (1) β-catenin expression is upregulated, (2) leading to its increased nuclear translocation. (3) In the nucleus, the β-catenin/p300 interaction is suggested to facilitate the acetylation (ac) of histone H3K27 at the promoters of specific genes, (4) supporting the transcription of *ITGB4* and laminin-332 subunit genes. (5) The resulting interaction between α6β4 integrin and laminin-332 activates Src signaling (phosphorylation), (6) ultimately promoting the malignant progression of PDAC. TF: Transcription factor

GEM resistance is associated with a poor prognosis in patients with pancreatic cancer [7]. Consistently, GEM treatment has been shown to induce malignancy in various human cancer cells by promoting tumorigenesis, invasion, and metastatic ability [31–33]. In this study, our data indicate that GEM-induced β4 integrin contributes to GEM resistance and cancer progression in PDAC cells. Specifically, our findings support a model in which nuclear β-catenin/p300 is associated with H3K27ac at the *ITGB4* promoter, thereby promoting β4 integrin expression at the epigenetic level. We therefore propose a model in which GEM treatment may paradoxically contribute to tumor progression by increasing β4 integrin expression, likely involving β-catenin/p300-mediated histone acetylation (Fig. 9). These findings reveal a compelling link between chemotherapy and the fundamental processes of cancer progression.

Notably, this regulatory axis is not a static event; our results suggest that it initiates a self-reinforcing positive feedback loop. Specifically, we observed that knockdown of β4 integrin in GEM-Panc-1 cells reduced the expression and nuclear translocation of β-catenin (Fig. S3A, β4 sh2). While re-introduction of WTβ4 integrin restored this level to baseline, the ADβ4 or Δcytoβ4 integrin mutants failed to do so (Fig. S3). These results suggest a positive feedback loop where β4 integrin–whose transcriptional activation is associated with β-catenin and epigenetic changes–further enhances β-catenin expression and its nuclear translocation, which in turn may sustain its own production.

Although the *ITGB4* promoter lacks canonical TCF/LEF-binding motifs, our findings show a strong co-localization of TCF family members, β-catenin, and p300 at this locus. This suggests that these factors form a functional transcriptional complex that contributes to *ITGB4* expression, likely through indirect recruitment of TCF/LEF proteins to the promoter. Consistent with this, silencing either β-catenin or p300 markedly reduced *ITGB4* levels and promoter activity. Furthermore, the enrichment of p300 correlates with high H3K27ac levels in GEM-Panc-1 cells, suggesting that the TCF/β-catenin/p300 complex is involved in the epigenetic activation of *ITGB4* transcription to promote PDAC progression.

The induction of specific integrins by chemotherapeutic agents is a recognized phenomenon in cancer biology [21]. In the present study, we showed that GEM treatment markedly induces β4 integrin expression in PDAC cells. Interestingly, this induction appears drug-specific; paclitaxel-resistant Panc-1 cells exhibited increased β4 integrin expression, whereas doxorubicin-resistant Panc-1 cells did not (Fig. S6). Similar observations have been reported in other malignancies; for instance, β4 integrin levels are substantially higher in tumor tissues from gefitinib-resistant gastric cancer patients compared to those from gefitinib-sensitive patients [34]. This adaptive upregulation extends to other integrin species as well. Although αvβ3 integrin was not substantially induced by GEM in our PDAC model (Fig. 3A), it is reportedly overexpressed in drug-resistant non-small cell lung cancer (NSCLC) cells [35]. Furthermore, β3 integrin-positive cells were identified in 97% of triple-negative breast cancer (TNBC) patient samples following chemotherapy, and increased β3 integrin-positive populations were observed in murine bone metastases after docetaxel treatment [36]. Collectively, these findings reinforce the notion that the chemotherapeutic induction of integrins is a context-dependent process, governed by both the specific agent and the cancer cell type.

Numerous preclinical studies have demonstrated that targeting αvβ3, αvβ5, or α5β1 integrins can inhibit tumor growth, metastasis, and angiogenesis in various cancers, including PDAC. However, despite these promising preclinical leads, clinical trial results have frequently fallen short of expectations [21]. This discrepancy may stem, in part, from the dynamic nature of integrin expression in tumor tissues, which can be markedly altered by prior chemotherapy and the emergence of chemoresistance–a phenomenon underscored by our current findings. Given that integrin expression patterns also evolve according to cancer stage [37], real-time assessment of the “integrin profile” in tumor tissue is likely essential for the success of anti-integrin therapies.

Chemotherapy modulates the tumor microenvironment (TME), which comprises the ECM and various non-cancerous cells, including myeloid cells, cancer-associated fibroblasts, and endothelial cells [10, 38, 39]. This remodeling of the TME may substantially contribute to the progression of GEM-resistant PDAC. Indeed, our study revealed that laminin-332–a primary ligand for β4 integrin–was upregulated in GEM-resistant PDAC cells, and its elevated expression correlated with reduced overall survival in PDAC patients. We further found that laminin-332 promotes the motility of GEM-resistant PDAC cells through its interaction with β4 integrin. These observations are supported by other studies indicating that the laminin γ2 chain promotes both cancer progression and GEM resistance in PDAC [40]. Notably, laminin-332 is produced not only by cancer cells but also by myofibroblasts and cancer-associated fibroblasts within the tumor stroma during tissue remodeling [41, 42]. Therefore, GEM-induced cancer progression may result from synergistic changes in both cancer cells and the TME, potentially characterized by the coordinated upregulation of β4 integrin and its ligand, laminin-332.

In this study, we identified that β4 integrin plays a pivotal role in mediating GEM resistance in PDAC cells. We previously reported that β4 integrin confers resistance to doxorubicin in Panc-1 and MDA-MB435S cells [43]. In contrast, a different study indicated that in TNBC cells, β4 integrin signaling through the DNA damage response pathway enhances sensitivity to cisplatin and carboplatin, while showing no effect on sensitivity to doxorubicin, GEM, or 5-FU [44]. These divergent findings suggest that the impact of β4 integrin on chemoresistance is highly context-dependent, varying from both cell type and the specific chemotherapeutic agent used. Given that multiple factors–including ECM, drug transporters, glycosylation, and EMT–are intertwined with drug resistance, the integrated output of these molecular players alongside β4 integrin likely determines the ultimate resistant phenotype [21, 33, 45, 46]. Further comprehensive investigations are warranted to fully elucidate the intricate molecular networks underlying GEM resistance.

Beyond its intracellular signaling, β4 integrin has been implicated in intercellular communication via extracellular vesicles. Specifically, the presence of β4 integrin in exosomes secreted by PDAC cells has been shown to increase exosome secretion and selectively promote lung metastasis [47]. Based on these reports and our current findings, it is plausible that GEM-resistant PDAC cells produce an abundance of β4 integrin-containing exosomes, potentially sustained by the identified positive feedback loop. Given that chemotherapy also increases the number of circulating tumor cells [48], both β4 integrin-enriched exosomes and β4 integrin-expressing circulating tumor cells in liquid biopsies may serve as valuable biomarkers. Such markers could be indicative of the emergence of GEM-resistant populations and the associated risk of disease progression.

It has been reported that PDAC cells undergo extensive chromatin remodeling during the metastatic process [49]. Our study suggests that GEM treatment epigenetically contributes to *ITGB4* expression, thereby promoting cancer progression. This finding suggests that chemotherapy can induce epigenomic alterations that further increase the diversity and complexity of cancer cell populations. Such change may accelerate intratumoral heterogeneity, contributing to the emergence of treatment-resistant clones and subsequent recurrence. Therefore, elucidating the molecular mechanisms underlying epigenetic-driven heterogeneity is a crucial step toward developing therapeutic strategies that effectively target resistant cancer cell populations. Our findings suggest that the β4 integrin/β-catenin positive feedback loop plays a pivotal role in maintaining GEM resistance and PDAC progression. Therefore, disrupting this signaling axis–perhaps through the development of small-molecule inhibitors or monoclonal antibodies targeting β4 integrin–could represent a promising clinical strategy. Integrating such targeted therapies with standard GEM-based chemotherapy may help overcome drug resistance and improve the prognosis of patients with advanced PDAC.

While these findings provide a strong rationale for clinical translation, certain limitations must be acknowledged. First, we did not perform a comprehensive analysis of genomic mutations or clonal evolution in GEM-resistant PDAC cells. It remains possible that underlying genetic alterations, in addition to the epigenetic mechanisms identified here, contribute to the observed phenotype. Future studies utilizing whole-exome sequencing or single-cell transcriptomics would be valuable to further elucidate the genomic landscape of GEM-resistant PDAC.

## Conclusions

Our study reveals a mechanism associated with GEM-induced β4 integrin expression in PDAC cells. Our findings support a model in which this upregulation involves β-catenin/p300-mediated histone acetylation at the *ITGB4* promoter, establishing a self-reinforcing positive feedback loop that may sustain drug resistance and subsequent disease progression. These results highlight β4 integrin as both a key mediator of GEM resistance and a promising biomarker for predicting clinical outcomes. Furthermore, our work provides a strong rationale for a new therapeutic paradigm: targeting the β4 integrin/laminin-332/Src kinase axis to circumvent GEM resistance and potentially halt cancer progression in PDAC. This inhibition strategy holds the potential to substantially improve the clinical outcomes of patients with GEM-resistant pancreatic cancer.

## Supplementary Information

Supplementary Material 1

## Data Availability

The datasets used and/or analyzed during the current study are available from the corresponding author upon reasonable request. The materials generated in this study can be requested from the corresponding author.

## Abbreviations

AD: Adhesion-deficient
CSC: Cancer stem cell
Ctrl: Control
Δcyto: Cytoplasmic domain-deleted
DMSO: Dimethyl sulfoxide
ECM: Extracellular matrix
EMT: Epithelial-to-mesenchymal transition
GEM: Gemcitabine
H3K27ac: Histone H3 lysine 27 acetylation
MET: Mesenchymal-to-epithelial transition
PDAC: Pancreatic ductal adenocarcinoma
TBST: TBS with 0.1% (v/v) Tween 20
TME: Tumor microenvironment
TNBC: Triple-negative breast cancer
WT: Wild-type
