# Supplementary material for: Gemcitabine-induced β4 integrin drives cancer progression and gemcitabine resistance in pancreatic cancer
Source: J Exp Clin Cancer Res. 2026 Apr 24;45:130. doi: 10.1186/s13046-026-03718-2 (PMC13244639; doi:10.1186/s13046-026-03718-2)
Supplement: Supplementary file 1 — Supplementary Material 1 [file 13046_2026_3718_MOESM1_ESM.pdf]

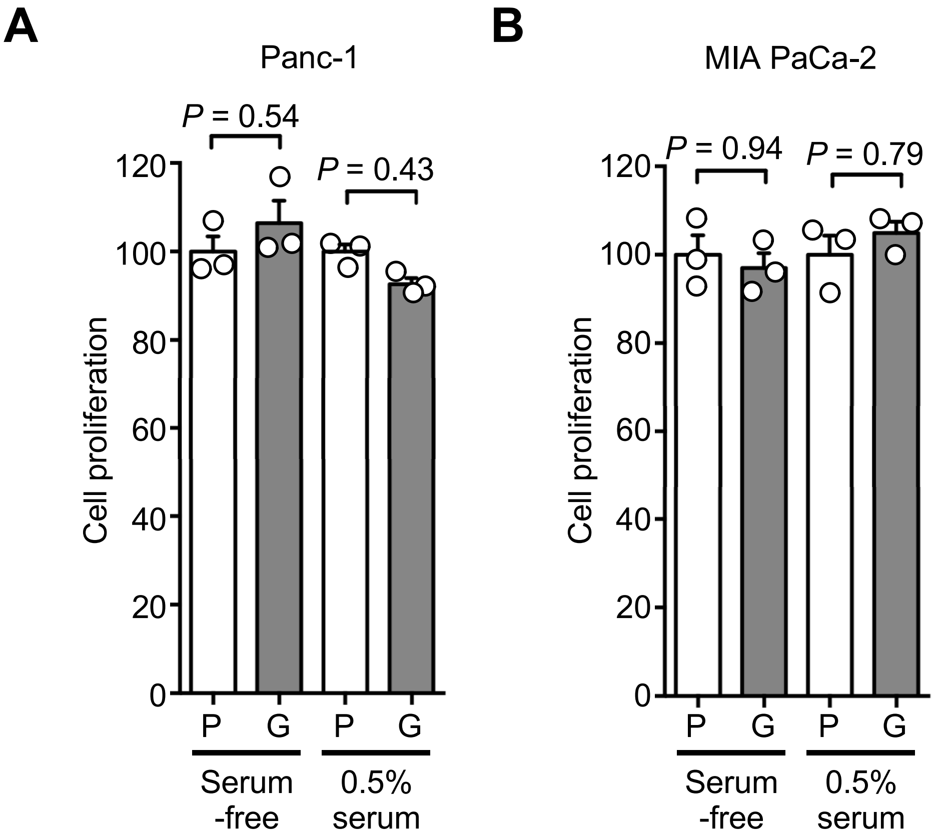

Fig. S1. Cell proliferation rates under Transwell assay conditions.
Panc-1 (1×10^4 cells/well) and MIA PaCa-2 (5×10^3 cells/well) cells were seeded in 96-well plates in 100 µL of medium with or without 0.5% FBS. **(A)** Relative proliferation rates of parental (P) and GEM-resistant (G) Panc-1 cells after 4 h of serum-free culture or 22 h of culture with 0.5% FBS. **(B)** Relative proliferation rates of parental (P) and GEM-resistant (G) MIA PaCa-2 cells after 22 h of culture with or without 0.5% FBS. Proliferation rates were normalized to the mean value of the corresponding parental cells, which was set to 100%. Data represent the mean ± SEM of three independent experiments performed in triplicate. Statistical significance was determined by a two-tailed unpaired Student's *t*-test.

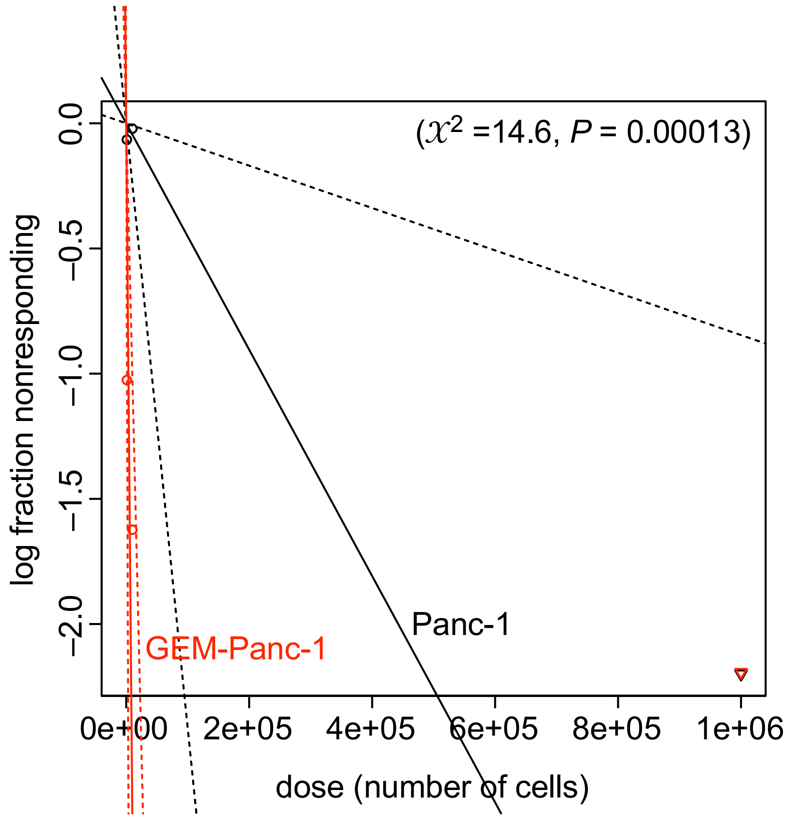

Fig. S2. *In vivo* limiting dilution assay of Panc-1 and GEM-Panc-1 cells. Mice were injected with the indicated number of cells (10^6, 10^4, 10^3). Log-fraction plot derived from ELDA. The estimated CSC frequency for GEM-Panc-1 was 1/3,596 (95% CI: 1/1,309 – 1/9,879), which was significantly higher than Panc-1 (1/221,200; 95% CI: 1/41,392 – 1/1,182,103) (P = 0.00013 by Chi-square test).

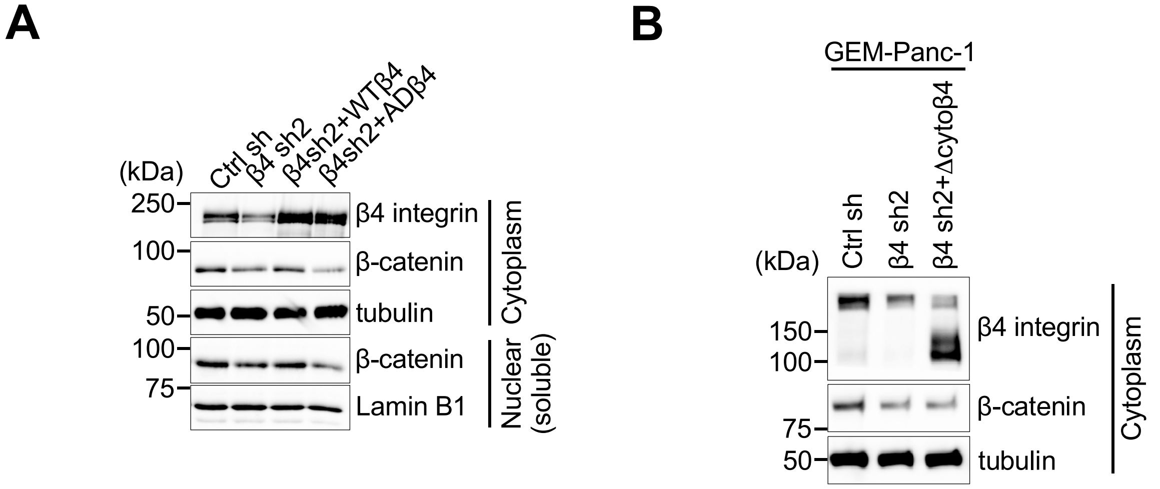

Fig. S3. Western blot analysis of β4 integrin and β-catenin expression in β4 integrin mutant-overexpressing GEM-Panc-1 cells. (A) Western blot analysis of β4 integrin and β-catenin in control (Ctrl) sh–, β4 sh2–, β4 sh2– and wild-type (WT) β4 integrin–, and β4 sh2– and adhesion -deficient (AD) β4 integrin–GEM-Panc-1 cells. **(B)** Western blot analysis of β4 integrin and β-catenin in control (Ctrl) sh–, β4 sh2–, β4 sh2– and cytoplasmic domain-deleted (Δcyto)–β4 integrin-overexpressing GEM-Panc-1 cells. Tubulin served as a loading control.

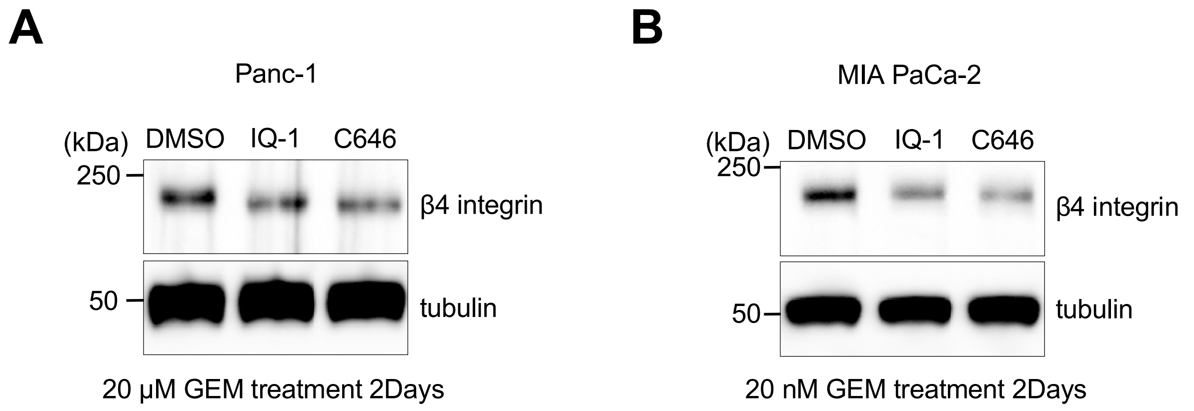

Fig. S4. Effects of IQ-1 and C646 on GEM-induced β4 integrin expression in Panc-1 and MIA PaCa-2 cells. Panc-1 and MIA PaCa-2 cells were pretreated with IQ-1 (40 μM) or C646 (40 μM) for 20 min, followed by treatment with 20 μM and 20 nM gemcitabine, respectively. After 48 h, cell lysates were collected and subjected to Western blot analysis for β4 integrin. Tubulin and DMSO were used as a loading control and vehicle control, respectively.

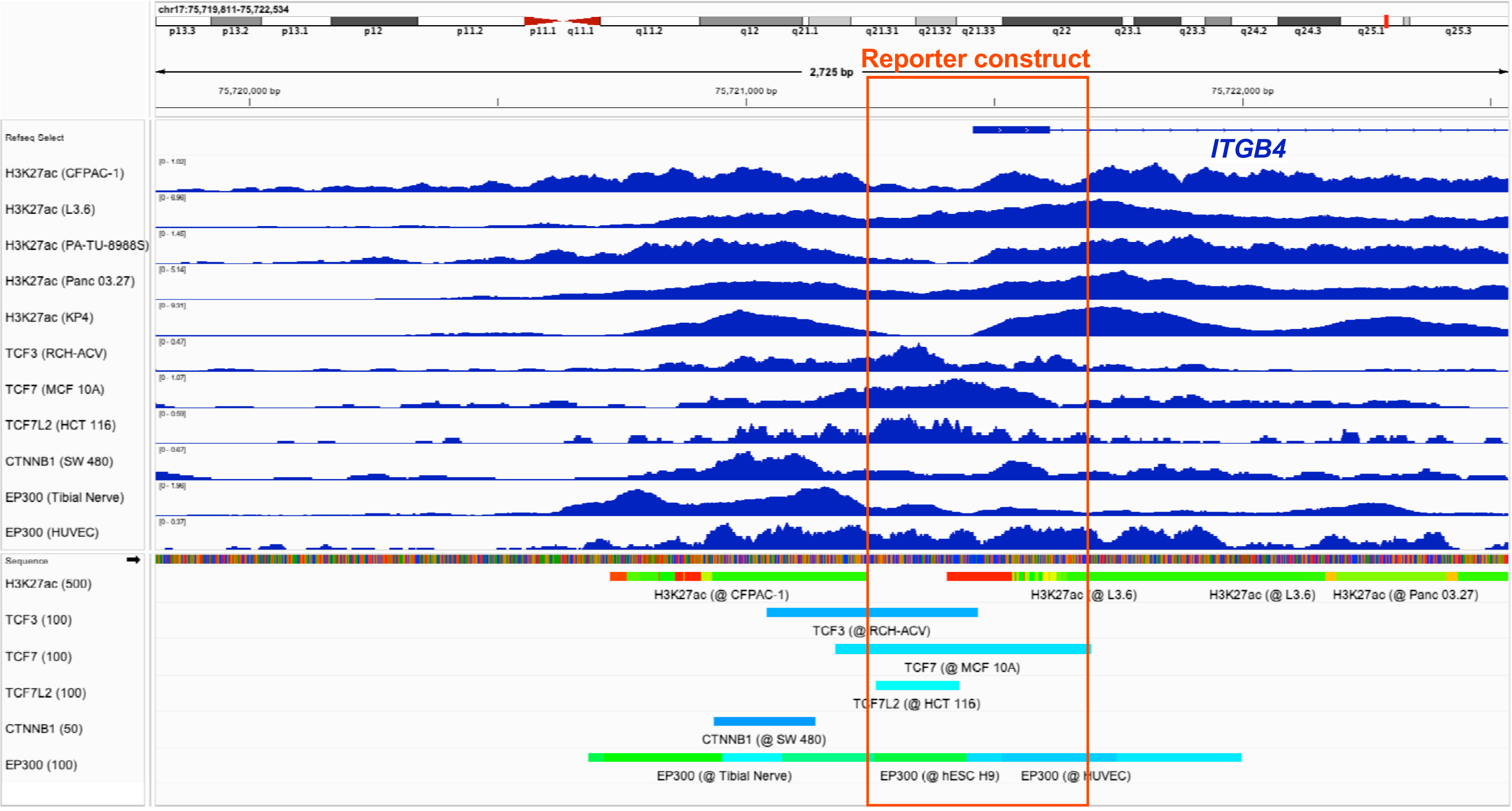

Fig. S5. Integrative genomic analysis of the *ITGB4* promoter and its regulatory landscape. IGV snapshots displaying ChIP-seq tracks for H3K27ac, Wnt-related transcription factors (*CTNNB1*, TCF family), and the co-activator *EP300* (p300) at the *ITGB4* locus. The red box indicates the specific promoter region used for the luciferase reporter assay.

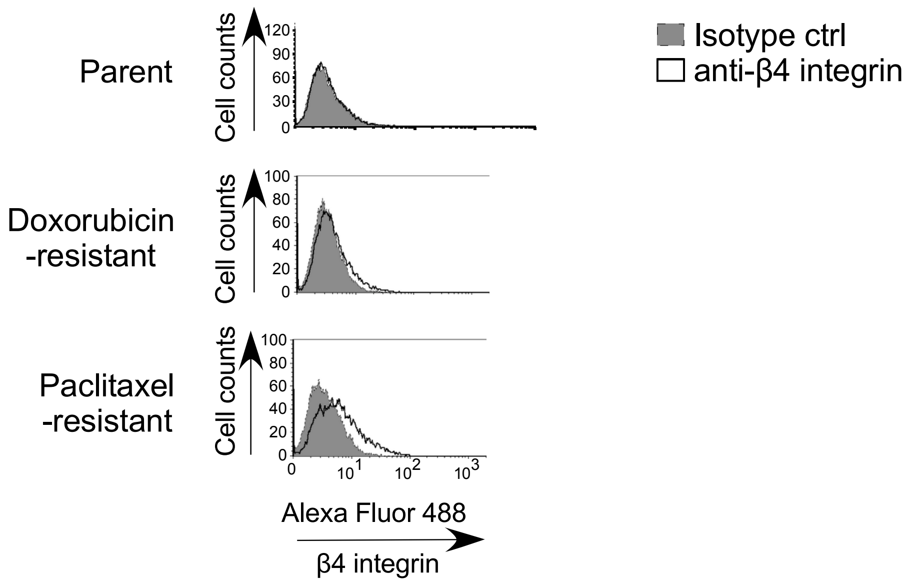

Fig. S6. Paclitaxel but not doxorubicin induces β4 integrin expression in Panc-1 cells. FACS analysis of cell surface expression of β4 integrin in doxorubicin- and paclitaxel-resistant Panc-1 cells.
